# Patient reported and functional outcome measures after surgical salvage procedures for posttraumatic radiocarpal osteoarthritis – a systematic review

**DOI:** 10.1186/s12891-024-07527-6

**Published:** 2024-06-07

**Authors:** Jane A. E. Gruisen, Philip M. J. Schormans, Ilona M. Punt, Alex K. Roth, Sander M. J. van Kuijk, Martijn Poeze, Pascal F. W. Hannemann

**Affiliations:** 1 Department of Trauma and Orthopedic Surgery, Maastricht University Medical Centre, PO Box 5800, 6202 AZ, Maastricht, The Netherlands; 2 Department of Surgery, Amphia Hospital Breda, Molengracht 21, 4818 CK Breda, The Netherlands; 3 Department of Klinische Epidemiologie en Medical Technology Assessment, Maastricht University, P.Debyelaan 25, Maastricht, 6229 HX The Netherlands

**Keywords:** Wrist osteoarthritis, Systematic review, Partial arthrodesis, Proximal row carpectomy

## Abstract

**Background:**

Posttraumatic wrist osteoarthritis is an irreversible and often progressive condition. Many surgical treatments, used in (daily) practice, aim to relieve symptoms like pain and restore function. The aim of this systematic review is to assess the patient reported and functional outcomes of the most common surgical interventions in patients with posttraumatic wrist osteoarthritis. This overview can help clinicians select the best treatment and manage patient’s expectations.

**Methods:**

A literature search was performed in Pubmed, Embase and Cochrane for articles published between 1990 and November 2022 according to the PRISMA guidelines. The study protocol has been registered in the PROSPERO database (CRD42017080427). Studies that describe patient reported outcomes (pain and Disability of Arm, Shoulder and Hand (DASH) –score) and functional outcomes (range of motion (ROM) and grip strength) after surgical intervention with a minimal follow-up of 1 year were included. The identified surgical procedures included denervation, proximal row carpectomy, interpositional- and total arthroplasty, and midcarpal-, radiocarpal- and total arthrodesis. The pre-and postoperative outcomes were pooled and presented per salvage procedure.

**Results:**

Data from 50 studies was included. Pain score improved after all surgeries except denervation. Flexion/extension decreased after radiocarpal arthrodesis, did not show significant changes after proximal row carpectomy, and improved for all other surgeries. DASH score improved after arthroplasty, proximal row carpectomy and midcarpal arthrodesis. Grip strength improved after interposition arthroplasty and partial arthrodesis.

**Conclusion:**

Evidence from this review did not support the indication for denervation in this particular patient population. In patients with SLAC/SNAC II, proximal row carpectomy might be favourable to a midcarpal arthrodesis solely based on better FE ROM of the radiocarpal joint after proximal row carpectomy. In terms of radiocarpal mobility, total wrist arthroplasty might be preferred to radiocarpal arthrodesis in patients with osteoarthritis after a distal radius fracture. More uniform measurements of outcomes would improve the understanding of the effect of surgical treatments of the posttraumatic osteoarthritic wrist.

**Supplementary Information:**

The online version contains supplementary material available at 10.1186/s12891-024-07527-6.

## Introduction

Posttraumatic wrist osteoarthritis is an irreversible and often progressive condition. It commonly occurs secondary to fractures of either the distal radius or the carpal bones or secondary to ligamentous disruption of the radiocarpal or intercarpal joints. [1–3]. At the radial side of the radiocarpal joint, osteoarthritis can occur secondary to a distal radius fracture in which the cartilage covering the radius is damaged [4, 5]. Although more frequently seen after intra-articular fractures, osteoarthritis can also occur after extra-articular fractures.

At the carpal side of the radiocarpal joint, wrist osteoarthritis occurs mostly secondary to either a scaphoid nonunion advanced collapse (SNAC) or a scapholunate advanced collapse (SLAC) of the proximal carpal row. [5–7] Both the scaphoid nonunion and scapholunate ligamentous injury potentially lead to alteration of the kinetics of the proximal carpal row. The altered biomechanics cause a predictive pattern of osteoarthritis, starting at the radial styloid and progressing to the scaphoid fossa and midcarpal joints. [6, 8, 9]

Osteoarthritis of the wrist can eventually lead to severe pain and functional impairment [9]. The primary goal of treatment is to decrease pain. The secondary goal is to preserve a functional range of motion and stability of the affected wrist [10]. Patients with radiocarpal osteoarthritis can frequently be helped by nonsurgical interventions. The use of immobilizing casts, non-steroidal anti-inflammatory drugs or intra-articular injections of corticosteroids may enable and improve functional use of the wrist by decreasing pain. Surgery is indicated only when non-surgical treatment fails [3, 6–8]. Numerous techniques have been proposed, including denervation, excisional arthroplasties, partial or total wrist arthrodesis and partial or total wrist arthroplasties. The choice of surgery depends heavily on the patient’s occupational and functional demands and the degree of wrist osteoarthritis [7, 8, 11].

Numerous retrospective single-centre cohort studies describing a plethora of surgical techniques for treating the osteoarthritic wrist have been published. Additionally, multiple systematic reviews and comparative studies describing outcomes after various wrist salvage procedures have been published [12–15]. However, these studies do not describe the outcomes exclusively for posttraumatic wrist osteoarthritis patients. Although the incidence of posttraumatic wrist osteoarthritis is unknown, it is becoming a more frequent and challenging problem in today’s demanding patient population. An overview of patient reported and functional outcomes per surgery could help manage a patient’s expectations and help select the best surgical treatment for each specific patient. Therefore, the aim of this study is to systematically review the literature on patient reported and functional outcomes for the most frequently described surgical techniques for treating posttraumatic wrist osteoarthritis.

## Methods

This study was performed according to Preferred Reporting Items for Systematic review and Meta-Analyses (PRISMA) [16]. The study protocol has been registered in the PROSPERO database (CRD42017080427).

## Data sources and searches

Electronic databases Pubmed, OVID Embase and Cochrane Central were systematically searched to find eligible studies covering January 1^st^ 1990 until November 15^th^ 2022. Studies published before 1990 were excluded since outcomes from surgical salvage procedures performed before 1990 only have historical value. Studies that described patient reported and functional outcomes after surgical salvage procedures for patients with posttraumatic wrist osteoarthritis with a minimal follow-up of 12 months were included. Studies written in a language other than English, Dutch, German or French were excluded. Furthermore, a manual search of the reference lists of the included full-text studies was performed. The detailed search strategy can be found in Additional Table S1.

## Eligibility criteria

Studies were included if 1) the patients were adults (18 years or older), 2) have undergone surgical salvage procedure for posttraumatic radiocarpal joint osteoarthritis (e.g., SNAC ≥II, SLAC ≥II, distal radius fracture) and 3) patient reported and functional outcome measures of interest (e.g., pain score (Visual Analogue Scale (VAS)), Disability Arm, Hand and Shoulder (DASH) score, flexion/extension (FE) and ulnar/radial deviation (RU) range of motion (ROM) and grip strength) with a minimal follow-up of 12 months were described.

Studies which included SNAC I, SLAC I or non-posttraumatic radiocarpal osteoarthritis such as rheumatoid arthritis or Kienbock’s disease were excluded if data of these patients could not be separated. Studies describing both posttraumatic radiocarpal osteoarthritis, secondary surgeries and non-posttraumatic radiocarpal osteoarthritis were only included when data were separately presented per patient (group). Finally, biomechanical and cadaveric studies, case studies (*n*<3), (systematic) reviews, meta-analyses and congress abstracts were excluded.

## Study selection

Eligible studies were imported into the Covidence systematic review software (Veritas Health Innovation, Melbourne Australia, available at www.covidence.org). Duplicates were removed by the Covidence software. Two reviewers (J.G., P.S.) independently evaluated titles and abstracts in a standardized blinded way. Inconsistencies were resolved by a consensus discussion. Uncertainty or unresolved disagreements were resolved by a third reviewer (I.P.). In case the title and abstract provided insufficient information to either in or exclude the study, the full-text was accessed and assessed for inclusion.

Next, eligible full-text studies were imported into the Covidence systematic review software and evaluated by two reviewers (J.G., P.S.).

## Data extraction

Three reviewers (J.G., P.S. or I.P.) independently extracted data using a standardized form created using Microsoft Access 2013 in a blinded manner. Inconsistencies were resolved in a consensus discussion. Uncertainty or unresolved disagreements were resolved by a third reviewer (P.S. or I.P.). The following information was collected: general study information (first author, year of publication, study design, country of study), type of surgical salvage procedure, participant characteristics at baseline (age, sex, diagnosis), patient reported and functional outcome measures of interest, unit of measurements and duration of follow-up. If data were missing, the corresponding author was contacted via e-mail (including one reminder). If the data was not provided, the study or a specific aspect of the study was excluded.

## Risk of *bias* assessment

The risk of bias of the included studies was independently evaluated by two reviewers (J.G., P.S.) using the validated Methodological Index for Non-Randomized Studies (MINORS) score (criteria 1-8) [17]. The items on the questionnaire were scored as not reported (0), reported but inadequate (1) or reported and adequate (2). With a maximum score of 16, the studies were rated as high (<8), moderate (8-12) or low risk of bias (>12). Conflicts in rating were resolved during a consensus meeting. A third reviewer (I.P.) was available to resolve any remaining disagreements.

## Data analysis

The outcomes of patient reported and functional measures were pooled using common-effects meta-analysis techniques stratified by surgery. Pre- and postoperative means were calculated for each functional outcome. As studies generally did not contribute head-to-head comparisons of pre-and postoperative scores, t-values and associated p-values were computed. As the correlation between pre- and postoperative scores was unknown, our test statistics can be regarded as conservative; actual p-values would be lower given a positive correlation between scores. A significance level of 0.05 and a confidence level of 0.95 were used. For patients with a SNAC or SLAC grade II, the most commonly performed surgical salvage procedure is proximal row carpectomy or midcarpal arthrodesis [18]. Multiple systematic reviews and meta-analyses have been performed to compare the outcomes of these surgical procedures. Some of these studies either failed to specify the degree of SLAC or SNAC or they included patients with a SLAC or SNAC grade III. Therefore, a sub-analysis of patients with SNAC or SLAC grade II after proximal row carpectomy and midcarpal arthrodesis is described separately.The pre-and postoperative difference in patient reported and functional outcomes were also compared with the Minimum Clinically Important Difference (MCID). The MCID is the smallest change in a treatment outcome that an individual patient would identify as important and which would indicate a change in the patient's management. If the found pre- and postoperative difference is larger than the MCID, we consider this difference clinically relevant. No MCID for the VAS score, DASH score and grip strength has been specifically described for wrist salvage procedures. To provide some perspective, we refer to MCID found in literature that was calculated in postoperative upper extremity populations. According to Randall et al., the MCID for the VAS score in a population undergoing non-shoulder, hand and upper extremity surgery ranges from 16 and 19 [19]. For the DASH score, the MCID is 10 according to Gummesson et al. in a population with patients who received surgery for upper extremity musculoskeletal conditions [20]. Additionally, Kim et al. presented a MCID for grip strength of 19.5% of the unaffected wrist in a patient population that received a volar plate fixation to treat a distal radius fracture [21].

## Results

### Search results

**Fig. 1:**
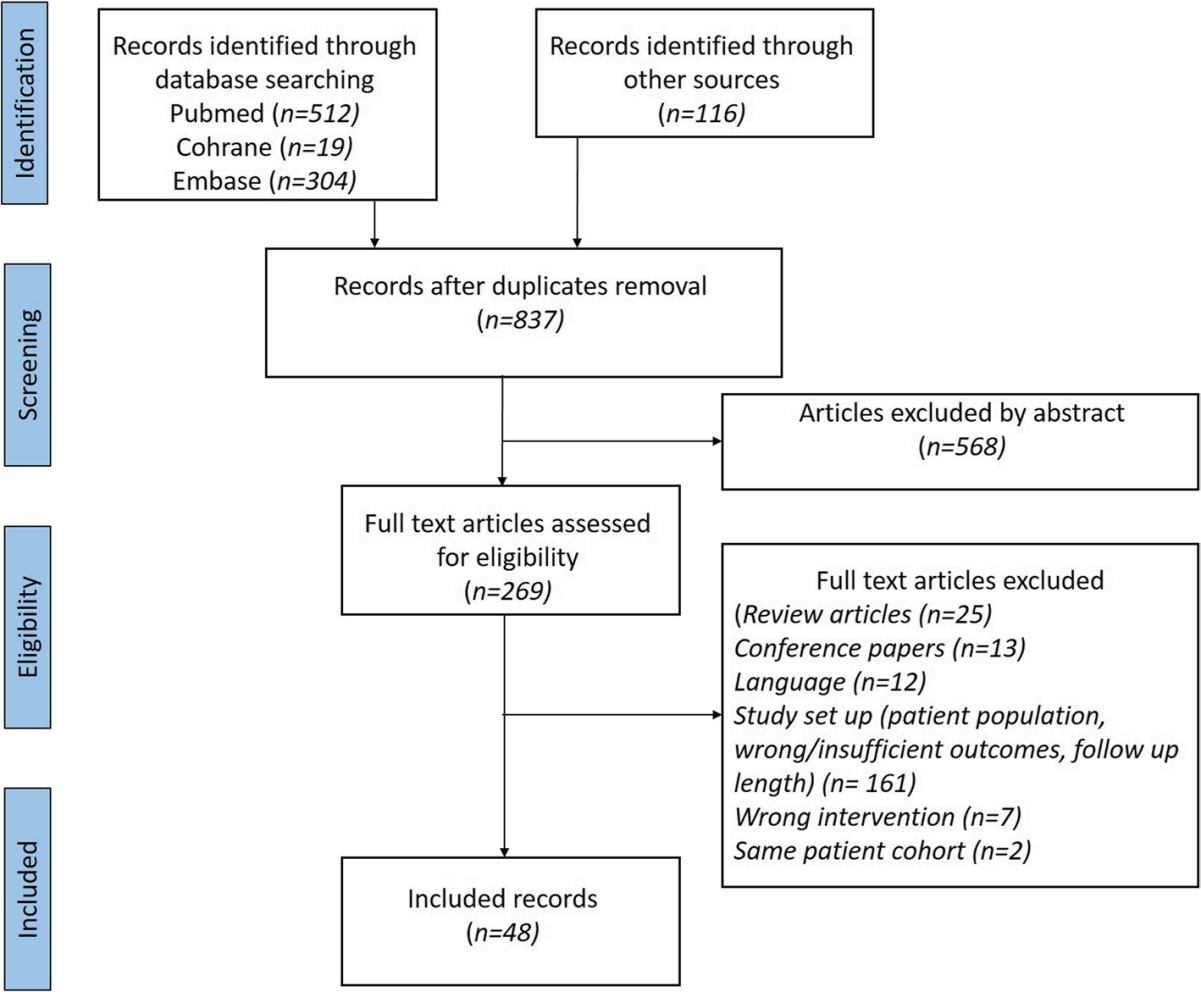
PRISMA flow chart illustrating the selection of studies included in the systematic review

A total of 951 studies were identified in all databases, which resulted in 837 studies for screening after duplicate removal. After evaluating the abstracts, 269 full text studies were examined and 50 studies were included in the systematic review (see Fig. 1).

### Study characteristics

The studies were divided by the performed surgery (denervation (*n*=4), proximal row carpectomy (*n*=10), interposition arthroplasty (*n*=2), total arthroplasty (*n*=2), total arthrodesis (*n*=1) and partial arthrodesis divided in midcarpal (*n*=25) and radiocarpal arthrodesis (*n*=10)). Characteristics and outcome measures of all studies per surgery can be found in Tables 1 and 2.

**Table 1:** Characteristics of included studies divided per surgical salvage procedure

**Study / country**	**Surgery type**	**Underlying pathology**	**n patients (n wrists)**	**Mean age (years) (SD, range)**	**Sex** **(n male: n female)**	**Mean follow-up (years) (SD, range)**
**Denervation**						
**Radu et al. 2010 / Germany ** [22]	wrist denervation (total/partial)	SLAC/SNAC *n=11*	30(30)	51.6 (34-69)^a^	33:10^a^	4.3 (1.5-8.1)^a^
		DRF or other trauma without carpal instability *n=19*				
**Rothe et al. 2006 / Germany ** [23]	(isolated) wrist denervation (Wilhelm)	SNAC II-III *n=36* SLAC II-III *n=10*	46(46)	47.0 (10.0, 26-76)	40:6	6.3 (2.3-14.0)
**Schweizer et al. 2006 / Switzerland ** [24]	total wrist denervation (Wilhelm)	SNAC *n=24(25)* SLAC *n=8* DRF *n=11*	43(44)	45.0 (14.0, 19-80)^a^	52(53):18^a^	9.6 (5.9, 1.0-23.0)^a^
**Weinstein et al. 2002 / USA ** [25]	AIN and PIN	SLAC II *n=5* SLAC IV *n=8(9)* DRF* n=1*	9(10)	53.6 (8.0, 45-63)	NR	2.2 (0.8, 1.1-3.7)
**Interposition arthroplasty**						
**Pequignot et al. 2000/ France ** [26]	APSI	SNAC II *n= 8* SNAC III *n= 3* SLAC II *n= 3* SLAC III *n= 2*	16 (16)	49.0 (8.1, 38-70)	24:1^a^	5.8 (2.5, 3.0-10.0)
**Szalay et al. 2011 / Germany ** [27]	RCPI with PRC	SNAC *n=1* SLAC *n=4*	5(5)	40.2 (17.2, 23-66)	3:2	4.5 (1.4, 2.1-5.6)
**Total arthroplasty**						
**Holzbauer et al. 2022 / Austria** [28]	TWA	SNAC *n=13*	13(13)	63.4 (8.0)	10:3	4.5 (2.9)
		SLAC *n=11*	11(11)	56.7 (9.9)	8:3	6.8 (3.3)
		DRF *n=8*	8(8)	57.5 (8.8)	2:6	7.4 (3.0)
**Reigstad et al. 2012 / Norway** [29]	Motec	SNAC III-IV *n=16* SLAC III-IV *n=14*	30 (30)	52.4 (31-71)	20:10	1.0 and 2.0
**Proximal row carpectomy**						
**Aita et al. 2016 / Brazil ** [30]	PRC	SNAC II	13(13)	32.4 (9.4, 18-52)	12:1	6.1 (0.3, 5.7-6.5)
**Cohen et al. 2001 / USA ** [31]	PRC	SLAC II	19(19)	48 (13.0, 32-73)	17:2	1.6 (1.0-3.0)
**Jebson et al. 2003 / USA ** [32]	PRC	SLAC *n=7* Scaphoid nonunion *n=10* Transcaphoid perilunate fracture dislocation *n=1*	18(18)	45.1 (16.6, 24-72)	NR	13.1 (10.0-17.2)
**Nagelvoort et al. 2002 / the Netherlands ** [33]	PRC	Scaphoid nonunion *n=3*	3(3)	36.3 (13.6, 28-52)	3:0	5.5 (4.9-6.2)
**Pogliacomi et al. 2014 / Italy ** [34]	PRC	SNAC II *n=4* SLAC II* n=6* SLAC III *n=2*	12(12)	59.3 (12.6, 40-80)	8:4	6.6 (6.1, 1.0-18.0)
**Salomon et al. 1996 / USA **[35]	PRC with partial resection capitate	SLAC III *n=6* Scaphoid malunion *n=1* Scaphoid nonunion *n=3*	10(10)	49.0 (31-66)^a^	10:0	4.6 (2.9, 1.1-10.2)
**De Smet et al. 2005 / Belgium ** [36]	PRC	SNAC *n=9* SLAC *n=17*	26(26)	48.0 (13.6)	22:4	5.5 (minimum 1.2)
**Streich et al. 2008 / Germany ** [37]	PRC	SNAC II *n=9* SLAC II *n=3*	12(12)	44.9 (15.5, 21-70)	12:0	[5.5] (2.8-11.8)^a^
**Midcarpal arthrodesis**						
**Abdelaziz et al. 2020 / Egypt ** [38]	CL arthrodesis with SE	SNAC III +17	15(15)	32.0 (20-37)	15:0	2.1 (1.7-3.0)
**Aita et al. 2016 / Brazil ** [30]	4CA	SNAC II	14(14)	40.4 (8.9, 25-56)	12:2	6.1 (0.4, 5.6-6.8)
**Calandruccio et al. 2000 / USA **[39]	CL arthrodesis	SNAC *n=3* SLAC *n=10* Perilunar fracture dislocation *n=1*	14(14)	49.0 (20-70)	14:0	2.3 (1.2-4.3)
**Cha et al. 2013 / Korea ** [40]	4CA	SLAC III	42(42)	46.9 (42-61)	33:9	12.2 (10.8-13.6)
**Chung et al. 2016 / USA ** [41]	4CA	SLAC II	11(11)	49.4 (35-65)	8:3	1.0
**Cohen et al. 2001 / USA ** [31]	4CA	SLAC II *n= 18* SLAC III *n=1*	19(19)	47.0 (15.0, 24-70)	17:2	2.3 (1.0-4.8)
**Le Corre et al. 2015 / France ** [42]	4CA plate	SNAC II *n=1* SNAC III *n=1* SLAC II *n=1* SLAC III *n=11*	15(15)	58.6 (47-70)	8:7	3.1 (1.9-6.7)
	4CA staple	SNAC II *n=3* SNAC III *n=4* SLAC III *n=30*	37(37)	58.5 (41-80)	31:6	4.3 (2.0-7.8)
**Dimitrios et al. 2010 / Greece ** [43]	CLS arthrodesis MCA	SNAC II *n= 3* SNAC III *n= 5*	8(8)	39.8 (7.8, 29-52)	8:0	4.3 (1.6, 2.3-6.9)
**Durand et al. 2007 / France** [44]	CL arthrodesis	SNAC II *n=5* SNAC III *n=4* SLAC II *n=1* SNAC III *n=1*	11(11)	53.4 (7.8, 45-73)	NR	3.9 (3.4, 1.0-11.0)
**Ferreres et al. 2009 / Spain ** [45]	CL arthrodesis	SNAC/SLAC	17(17)	46.7 (26-66)	12:5	10.4 (6.2-16.5)
**Ghargozloo et al. 2002 / Italy ** [46]	CL arthrodesis	SNAC II-III *n=3* SLAC II-III *n=3*	5(6)	55.0 (27-74)	6:0	2.0
**Hernekamp et al. 2016 / Germany ** [47]	4CA k-wire	SNAC II *n=1* SNAC III *n=6* SLAC III *n=3*	10(10)	46.6 (31-73)	9:1	4.5
	4CA plate	SNAC III *n=6* SLAC III *n=5*	11(11)	45.5 (24-70)	8:3	1.3
**Huang et al. 2021 / Taiwan ** [48]	CL arthrodesis	SNAC III *n=3* SLAC II *n= 2* SNAC III *n= 5*	10(10)	54.2 (13.5, 33-76)	7:3	1.7 (1.0-3.2)
**Kendal et al. 2005 / Kendall** [49]	4CA	SLAC II *n=2* SLAC III *n=1*	3(3)	48.7 (4.5, 44-53)	3:0	1.5 (0.1, 1.3-1.6)
**Khan et al. 2013 / UK ** [50]	4CA	SNAC *n=3* SLAC *n=5*	8(8)	55.7 (12.6. 37-69)	5:3	1.5 (0.4)
**Maire et al. 2011 / France ** [51]	4CA	SNAC III *n=5* SLAC III *n=4*	9(9)	51.9 (26-76)	8:1	2.3 (1.3-3.7)
**Mantovani et al. 2010 / Brazil ** [52]	4CA	SNAC II-III/SLAC	17(17)	42.2 (9.4, 25-59)	15:2	1.8 (1.2-2.6)
**Schindelar et al. 2022 / USA ** [53]	4CA	SNAC/SLAC	21(21)	54.0	6:15	8.8 (5.1-13.2)
**Singh et al. 2015/ UK**	3CA	SNAC II-III/SLAC II-III	12(12)	55.0 (10.0)	8:4	6.0
**De Smet et al. 2006 / Belgium ** [54]	4CA	SNAC *n=7* SLAC *n=11*	18(18)	56.0 (29-73)	14:4	2.6 (2.0)
**De Smet et al. 2009 / Belgium ** [55]	4CA	SNAC *n=8* SLAC *n=20*	28(28)	55.2 (28-74)	NR	2.4 (1.0-6.0)
**Tielemans et al. 2017 / Belgium ** [56]	4CA	SNAC II *n=5* SNAC III *n=4* SLAC II *n=3* SLAC III *n=9*	21(21)	[53.0] (28-78)	15:6	[1.3] (1.0-2.3)
**Undurraga et al. 2021 / Canada ** [57]	bicolumnar carpal fusion	SNAC II/III *n= 9* SLAC II/III *n=16*	23(25)	54.0	16:7	1.5 (1.1-2.7)
**Winkler et al. 2010 / Germany ** [58]	4CA	SNAC II *n= 4* SNAC III *n=3* SLAC III *n=5*	11(12)	NR	11:0	5 (3.3-6.3)
**Yao et al. 2017 / Taiwan ** [59]	CL arthrodesis	SNAC II/III *n=6* SLAC II/III *n=4*	10(10)	55.9 (7.9, 41-71)	10:0	3.7 (1.5, 1.8-5.7)
**Radiocarpal arthrodesis**						
**Bach et al. 1991 / USA ** [60]	RSL	SL dissociation *n= 8* Scaphoid nonunion *n=1* DRF *n=2* DJD *n=1*	12(12)	51.0 (9.7, 39-72)	11:2	3.1 (1.7, 1.3-6.0)
**Beyermann et al. 2000 / Germany** [61]	RSL	DRF	10(10)	47.7 (14.2, 27-73)	7:3	2.3 (1.4, 1.0-5.5)
**Degeorge et al. 2020 / France ** [62]	RSL	DRF malunion *n=25* Dislocation DRF *n=4*	29(29)	54.0 (12.0)	24:5	8.9 (7.2)
	RSL + DSE	SLAC II *n=1* DRF malunion *n=20* Dislocation DRF *n=2*	23(23)	48.0 (14.0)	20:3	8.0 (5.9)
	RSL + DSE + TE	SLAC II *n=1* DRF malunion *n=9* Dislocation DRF *n=2*,	12(12)	56.0 (11.0)	10:2	11.7 (6.6)
**Garcia-Elias et al. 2005 / Spain ** [63]	RSL+DSE	DRF *n=13* Perilunate fracture dislocation *n=2*	15(15)	41.1 (14.6, 18-64)	12:3	2.8 (1.6, 1.0-5.8)
**Inoue et al. 1992 / Japan** [64]	RL/RSL	DRF	5(5)	34.8 (13.5, 23-56)	5:0	3.4 (0.7, 2.6-4.2)
**Kilgus et al. 2003 / Switzerland ** [65]	RSL	SL dissociation *n=1* Scaphoid pseudoarthritis *n=2* DRF *n=1*,	4(4)	37.5 (25-46)^a^	NR	17.8 (15.8-22.0)^a^
**Quadlbauer et al. 2017 / Austria **[66]	RSL+DSE	DRF malunion	11(11)	55.0 (16.0, 35-86)	7:4	5.1 (1.3, 2.5-8.1)
**Tomaino et al. 1994 / USA** [67]	RL/RSL	PT	3(3)	NR	3:0	3.9 (2.2-5.5)
**Yajima et al. 1994 / Japan **[68]	RL/RSL	DRF	5(5)	43.6 (6.5)	1:4	3.1 (0.5, 2.5-3.8)
**Yajima et al. 2004 / Japan **[69]	RL/RSL	DRF	9(9)	41.4 (23-70)	8:1	8.5 (2.0-33.0)
**Total arthrodesis**						
**De Smet 2006/ Belgium ** [54]	Total arthrodesis	SNAC *n=5* SLAC *n=14*	19(19)	49.0 (32-69)	10:9	5.3 (0.7)

^a^mean scores including excluded patients

*AIN * anterior interosseous neurectomy, *PIN* posterior interosseous neurectomy, *PRC* proximal row carpectomy, *RCPI* resurfacing capitate pyrocarbon implant, *APSI* adaptive proximal scaphoid implant, Total wrist arthroplasty, *CL* capitolunate arthrodesis, *3CA* three-corner arthrodesis, *4CA* four-corner arthrodesis, *RL* radiolunate arthrodesis, *RSL* radioscapholunate arthrodesis, (*D*)*SE* (distal) scaphoid excision, *TE* triquetrum excision

*SNAC* scaphoid nonunion advanced collapse, *SLAC* scapholunate advanced collapse, *DRF* distal radial fracture, *DJD* degenerative joint disease, *PT* posttraumatic arthritis

[] median

**Table 2:** Patient reported and functional outcome measures of included studies divided per salvage procedure

Study	N		ROM FE (degrees)		ROM RU (degrees)		Strength (% of unaffected wrist)		VAS score		DASH score	
	**Pre**	**Post**	**Pre**	**Post**	**Pre**	**Post**	**Pre**	**Post**	**Pre**	**Post**	**Pre**	**Post**
Denervation												
Radu et al. 2010 [22]	11(11)	11(11)	96.8	78.5	42.0	44.5	NR	NR	71.0	64.0	NR	37.8
	19(19)	19(19)	81.2	69.4	39.1	42.5	NR	NR	70.0	59.0	NR	45.5
Rothe et al. 2006 [23]	46(46)	46(46)	86.3	79.4	35.4	29.2	46.0	70.0	68.1	25.6	NR	17.1
Schweizer et al. 2006 [24]	24(25)	24(25)	NR	NR	NR	NR	NR	NR	NR	NR	NR	22.3
	8(8)	8(8)									NR	20.3
	11(11)	11(11)									NR	32.9
Weinstein et al. 2002 [25]	9(10)	9(10)	NR	NR	NR	NR	NR	NR	NR	NR	NR	34.2
Interposition arthroplasty												
Pequignot et al. 2000 [26]	16(16)	16(16)	NR	93.9	NR	NR	NR	81.3	NR	31.20	NR	NR
Szalay et al. 2011 [27]	5(5)	5(5)	54.0	79.0	28.0	32.0	70.0	88.0	6.1	1.4	51.3	8.0
Total arthroplasty												
Holzbauer et al. 2022 [28]	13(13)	13(13)	52.0	77.0	27.0	40.0	NR	76.9	70.0	22.0	62.0	24.0
	11(11)	11(11)	50.0	63.0	21.0	35.0	NR	70.3	70.0	31.0	60.0	31.0
	8(8)	8(8)	39.0	56.0	21.0	29.0	NR	52.3	69.0	36.0	69.0	35.0
Reigstad et al. 2012 [29]	27(27)	27(27)	NR	NR	NR	NR	63.0	63.0	7.4	1.9	43.0	19.2
	21(21)	21(21)^a^	NR	NR	NR	NR	63.0	79.0	7.4	2.1	43.0	17.5
Proximal row carpectomy												
Aita et al. 2016 [30]	13(13)	13(13)	80.5	108.9	NR	NR	NR	78.7	7.5	2.3	NR	11.0
Cohen et al. 2001 [31]	19(19)	19(19)	NR	81.0	NR	31.0	NR	71.0	7.2	1.4	NR	NR
Jebson et al. 2003 [32]	18(18)	18(18)	84.4	75.3	38.7	34.6	NR	90	NR	NR	NR	NR
Nagelvoort et al. 2002 [33]	3(3)	3(3)	NR	79.4	NR	45.3	NR	91.8	NR	0	NR	14.0
Pogliacomi et al. 2014 [34]	12(12)	12(12)	NR	NR	NR	NR	NR	NR	NR	NR	51.9	13.6
Salomon et al. 1996 [35]	10(10)	10(10)	80.0	90.5	NR	39.5	NR	NR	NR	NR	NR	NR
De Smet et al. 2005 [36]	26(26)	26(26)	NR	101.0	NR	NR	NR	73.5	NR	NR	NR	16.0
Streich et al. 2008 [37]	12(12)	12(12)	51.3	77.9	21.3	33.3	NR	73.6	NR	NR	NR	30.0
Midcarpal arthrodesis												
Abdelaziz et al. 2020 [38]	15(15)	15(15)	64	70.9	NR	NR	NR	71.5	NR	NR	NR	NR
Aita et al. 2016 [30]	14(14)	14(14)	50.6	118.4	NR	NR	NR	65.4	8.2	2.9	NR	13.0
Calandruccio et al. 2000 [39]	14(14)	14(14)	NR	53.0	NR	18.0	NR	71.7	NR	NR	NR	NR
Cha et al. 2013 [40]	42(42)	40(40)	83.5	65.9	45.4	39.1	70.5	85	6.3	2.0	43.8	16.8
Chung et al. 2016 [41]	11(11)	10(10)	86.9	73.8	48.3	37.2	NR	NR	53.6	42.0	NR	NR
Cohen et al. 2001 [31]	19(19)	19(19)	NR	80.0	NR	53.0	NR	79.0	81.0	12.0	NR	NR
Le Corre et al. 2015 [42]	15(15)	15(15)	66.5	58.9	27.0	25.1	NR	NR	57.3	14.5	58.0^a^	34.8^a^
	37(37)	37(37)	80.6	67.7	35.0	30.9	NR	NR	54.3	23.9	59.0^a^	39.4^a^
Dimitrios et al. 2010 [43]	8(8)	8(8)	73.4	83.5	41.5	50.1	NR	NR	NR	NR	NR	NR
Durand et al. 2007 [44]	11(11)	11(11)	59.5	60.5	NR	NR	60.0	54.0	NR	NR	NR	NR
Ferreres et al. 2009 [45]	17(17)	17(17)	84.0	77.0	32.0	39.0	NR	65.0	NR	NR	NR	7.7
Ghargozloo et al. 2002 [46]	5(6)	5(6)	NR	NR	NR	NR	NR	NR	NR	58.6	58.6	7.5
Hernekamp et al. 2016 [47]	10(10)	10(10)	NR	57.0	NR	30.0	NR	61.9	NR	[4.3]	NR	26.1
	11(11)	11(11)	NR	52.7	NR	30.5	NR	56.2	NR	[3.5]	NR	32.9
Huang et al. 2021 [48]	10(10)	10(10)	76.5	74.0	42.5	47.5	NR	NR	58.0	9.0	55.9	26.1
Kendal et al. 2005 [49]	3(3)	3(3)	NR	64.7	NR	NR	NR	53.3	NR	NR	NR	NR
Khan et al. 2013 [50]	8(8)	8(8)	NR	56.0	NR	26.5	NR	NR	NR	NR	NR	23.0^a^
Maire et al. 2011 [51]	9(9)	9(9)	92.2	59.4	NR	NR	54.9	69.8	5.78	1.22	47.2	16.9
Mantovani et al. 2010 [52]	17(17)	17(17)	31.1	77.1	17.9	34.4	35.8	81.6	NR	NR	89.5	42.4
Schindelar et al. 2022 [53]	21(21)	21(21)	NR	65.0	NR	NR	NR	92.0	NR	NR	NR	NR
Singh et al. 2015 [70]	12(12)	12(12)	NR	62.0	NR	38	NR	82.0	NR	[14.0]	NR	NR
De Smet et al. 2006 [54]	18(18)	18(18)	NR	84.0	NR	NR	66.7	67.0	NR	NR	NR	38.7
De Smet et al. 2009 [55]	28(28)	28(28)	73.0	51.0	NR	32.0	79.4	70.6	NR	NR	39	38
Tielemans et al. 2017 [56]	21(21)	21(21)	NR	NR	NR	NR	NR	53.0	6.3	2.9	54.1^a^	34.6^a^
Undurraga et al. 2021 [57]	23(25)	23(25)	NR	77.0	NR	43	NR	92.9	NR	NR	NR	19.0
Winkler et al. 2010 [58]	11(12)	11(12)	76.3	75.0	33.8	37.5	57.6	89.5	7.4	1.4	NR	15
Yao et al. 2017 [59]	10(10)	10(10)	61	72.5	NR	NR	NR	NR	5.0	1.1	NR	NR
Radiocarpal arthrodesis												
Bach et al. 1991 [60]	12(12)	12(12)	NR	44.3	NR	21.7	NR	NR	NR	NR	NR	NR
Beyermann et al. 2000 [61]	10/10	10(10)	76	48.0	32.5	26.0	NR	NR	70	41	NR	30.3
Degeorge et al. 2020 [62]	29(29)	29(29)	NR	49.0	NR	30.0	NR	NR	NR	NR	NR	42.7^b^
	23(23)	23(23)	NR	55.0	NR	32.0	NR	NR	NR	NR	NR	35.7^b^
	12(12)	12(12)	NR	59.0	NR	31.0	NR	NR	NR	NR	NR	34.8^b^
Garcia-Elias et al. 2005 [63]	FE: 14(14) RU: 13(13)	FE: 14(14) RU: 13(13)	68.2	64.6	26.2	33.4	NR	NR	NR	NR	NR	NR
Inoue et al. 1992 [64]	5(5)	5(5)	70.0	57.0	NR	25.6	30.6	70.8	NR	NR	NR	NR
Kilgus et al. 2003 [65]	4(4)	4(4)	NR	53.8	NR	30.0	NR	NR	NR	NR	NR	NR
Quadlbauer et al. 2017 [66]	11(11)	11(11)	64.0	95.0	NR	35.0	NR	80.0	NR	4.0	NR	39.0
Tomaino et al. 1994 [67]	3(3)	3(3)	NR	55.0	NR	NR	NR	66.3	NR	2.2	NR	27.0
Yajima et al. 1994 [68]	5(5)	5(5)	48.0	54.0	NR	NR	NR	NR	NR	NR	NR	NR
Yajima et al. 2004 [69]	9(9)	9(9)	66.3	62.8	NR	NR	NR	NR	NR	NR	NR	NR
Total arthrodesis												
De Smet et al. 2006 [55]	19(19)	19(19)	NR	NR	NR	NR	43.3	66.7	NR	NR	NR	45.2

Gray values do not have reported standard deviations and are not included in the analysis

^a^2 year follow up

^b^QuickDASH score

[] median

*ROM* range of motion, *FE* flexion/extension, *RU* radial/ulnar deviation, *VAS* visual analogue scale, *DASH* Disability of arm, shoulder, hand, *NR* not reported

### Risk of *bias*

All included studies demonstrated a moderate or low risk of bias. The scores for each individual study can be found in Additional Table S2.

### Participants

The diagnoses of the included patients per surgery are presented in Additional Table S3. Proximal row carpectomy, interposition arthroplasty and midcarpal arthrodesis were mainly performed in patients with SNAC/SLAC with a grade II/III. Total wrist arthroplasty was performed in both grade III or IV SNAC/SLAC and secondary to distal radius fractures. Radiocarpal arthrodesis was mainly performed in osteoarthritic wrists secondary to distal radius fractures. Denervation was performed in all of the above-mentioned diagnoses.

### Patient reported and functional outcomes

The pooled pre- and postoperative data, number of included studies and wrists per surgical procedure can be found in Additional Table S4. The MCID and difference between pre-and postoperative mean of VAS score, DASH score and grip strength are presented in Table 3.

**Table 3:** MCID and differences between pre and post surgery weighted mean of patient reported and functional outcomes

**Surgery**	**VAS score**	**DASH score**	**Grip strength**
			% of unaffected wrist
**MCID**	16-19	10-11	19.5
**Denervation**	-9.7	NR	NR
**Interposition arthroplasty**	-46.9	-43.3	14.1
**Total arthroplasty**	-41.5	-34.6	NR
**Proximal row carpectomy**	-58.3	-33.9	NR
**Midcarpal arthrodesis**	-43.3	-24.6	26.2
**Radiocarpal arthrodesis**	-41.5	NR	39.6
**Total Arthrodesis**	NR	NR	NR
**Weighted mean of patients suffering from SNAC or SLAC grade II**			
**Proximal row carpectomy**	-58.3	-29.3	NR
**Midcarpal arthrodesis**	-72.9	-22.7	1.2

*VAS* visual analogue scale, *DASH* Disability of arm, shoulder, hand, *NR* not reported

### Denervation

**Fig. 2:**
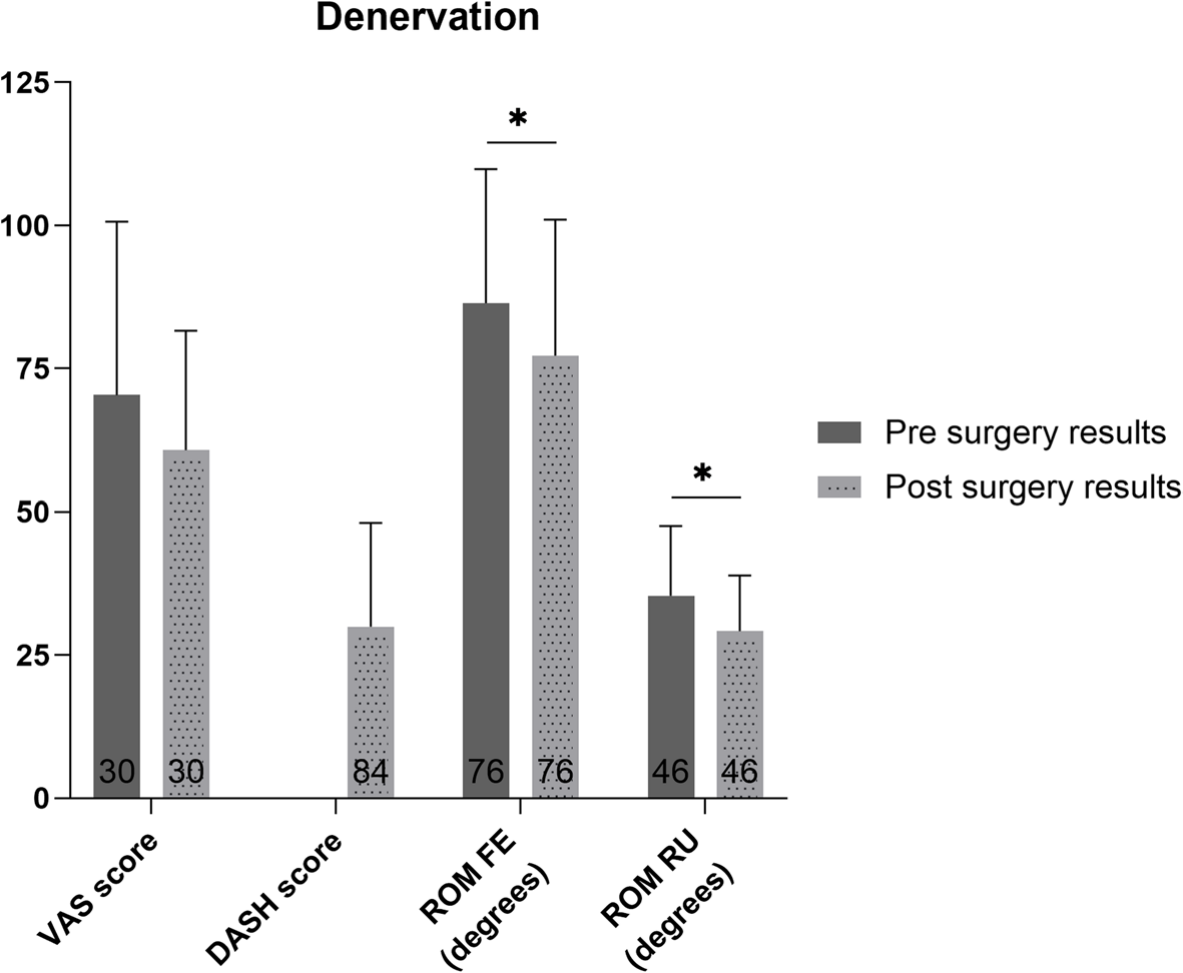
Pre- and postoperative weighted mean (with standard deviation) of patient reported and functional outcome measures for patients who underwent denervation. Number of patients on which the mean is calculated is depicted in the base of each bar

The weighted mean ROM FE and RU decreased (*p*<0.02), and the VAS score showed no significant change after denervation (*p*>0.05) (Fig. 2). No grip strength and only postoperative DASH scores were reported.

### Interposition arthroplasty

**Fig. 3:**
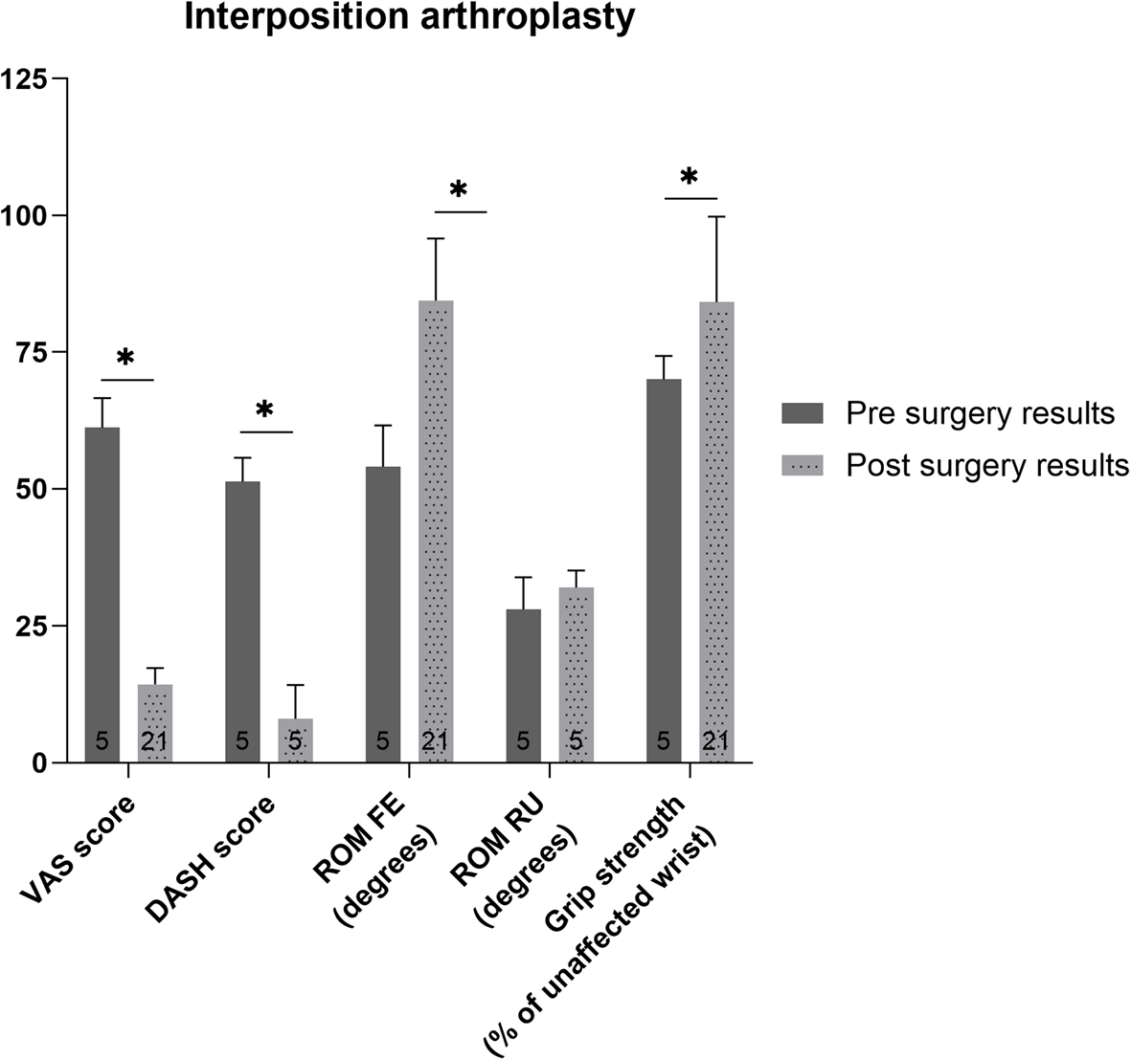
Pre- and postoperative weighted mean (with standard deviation) of patient reported and functional outcome measures for patients who underwent interposition arthroplasty. Number of patients on which the mean is calculated is depicted in the base of each bar

All patient reported and functional outcome weighted means improved (*p*<0.01), except for ROM RU which showed no significant change (*p*>0.05) after interposition arthroplasty (Fig. 3). The differences in pre- and postoperative patient reported outcomes were clinically relevant. However, the difference in pre-and postoperative grip strength was not clinically relevant.

### Total arthroplasty

**Fig. 4:**
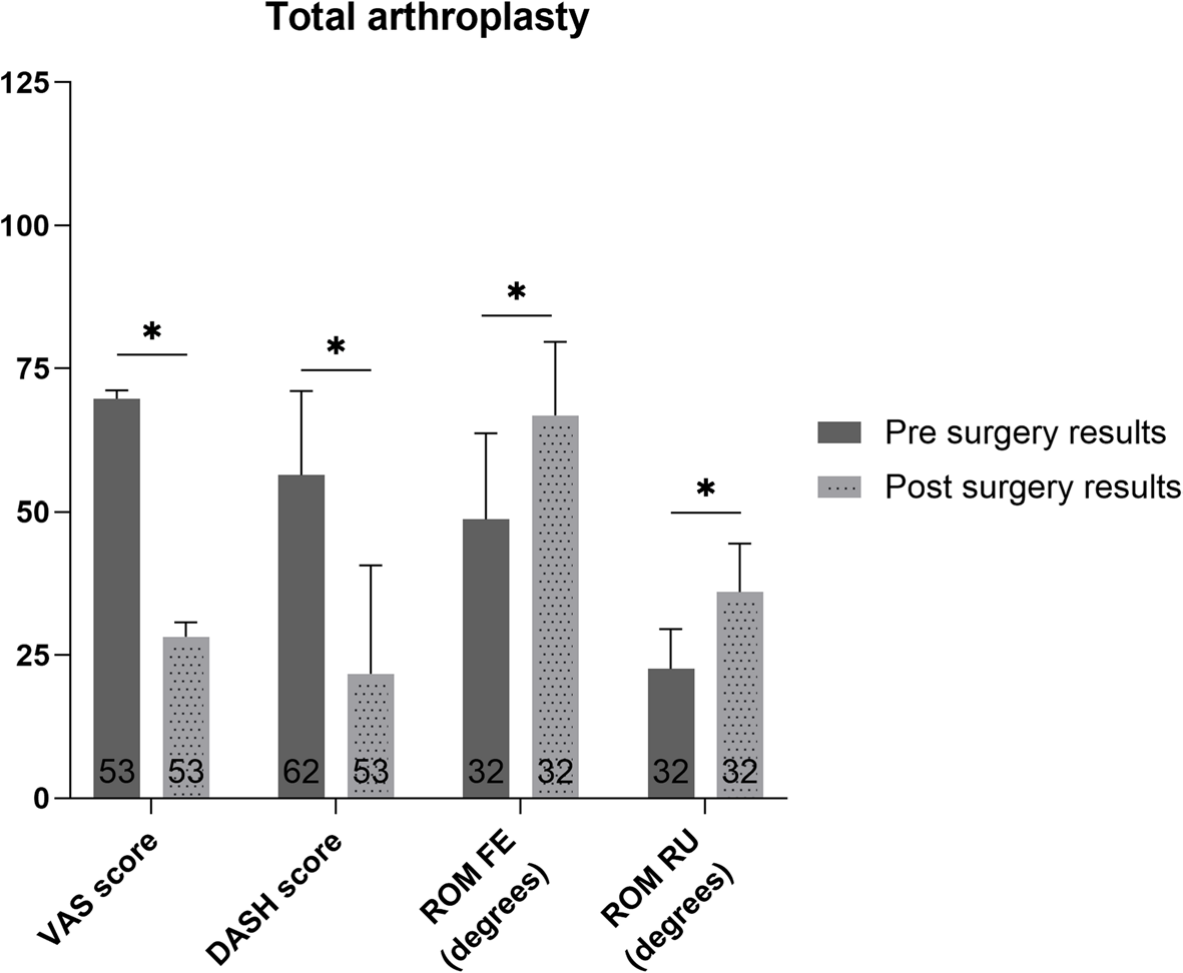
Pre- and postoperative weighted mean (with standard deviation) for patient reported and functional outcome measures of patients who underwent total arthroplasty. Number of patients on which the mean is calculated is depicted in the base of each bar

The weighted mean of the patient reported outcomes both decreased (*p*<0.01) and are clinically relevant (Fig. 4). The weighted mean ROM FE and RU increased (*p*<0.01). There were no DASH scores available.

### Proximal row carpectomy

**Fig. 5:**
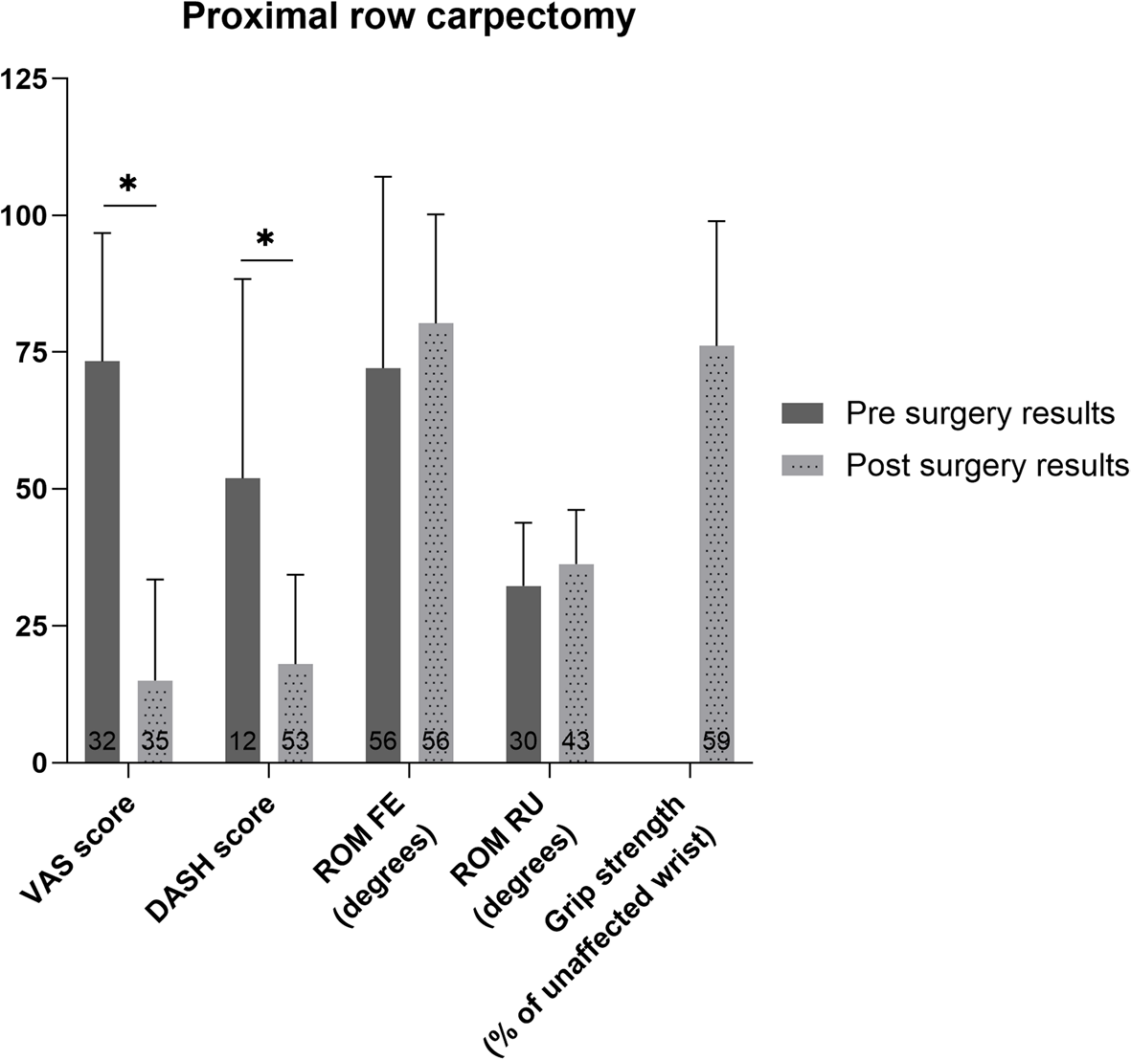
Pre- and postoperative weighted mean (with standard deviation) of patient reported and functional outcome measures for patients who underwent proximal row carpectomy. Number of patients on which the mean is calculated is depicted in the base of each bar

The weighted mean VAS score and DASH score improved after proximal row carpectomy (*p*<0.01) (Fig. 5). The difference in pre- and postoperative patient reported outcomes was clinically relevant. Additionally, the ROM FE and RU showed no significant change (*p*>0.05). Only post-surgery grip strength was reported.

### Midcarpal arthrodesis

**Fig. 6:**
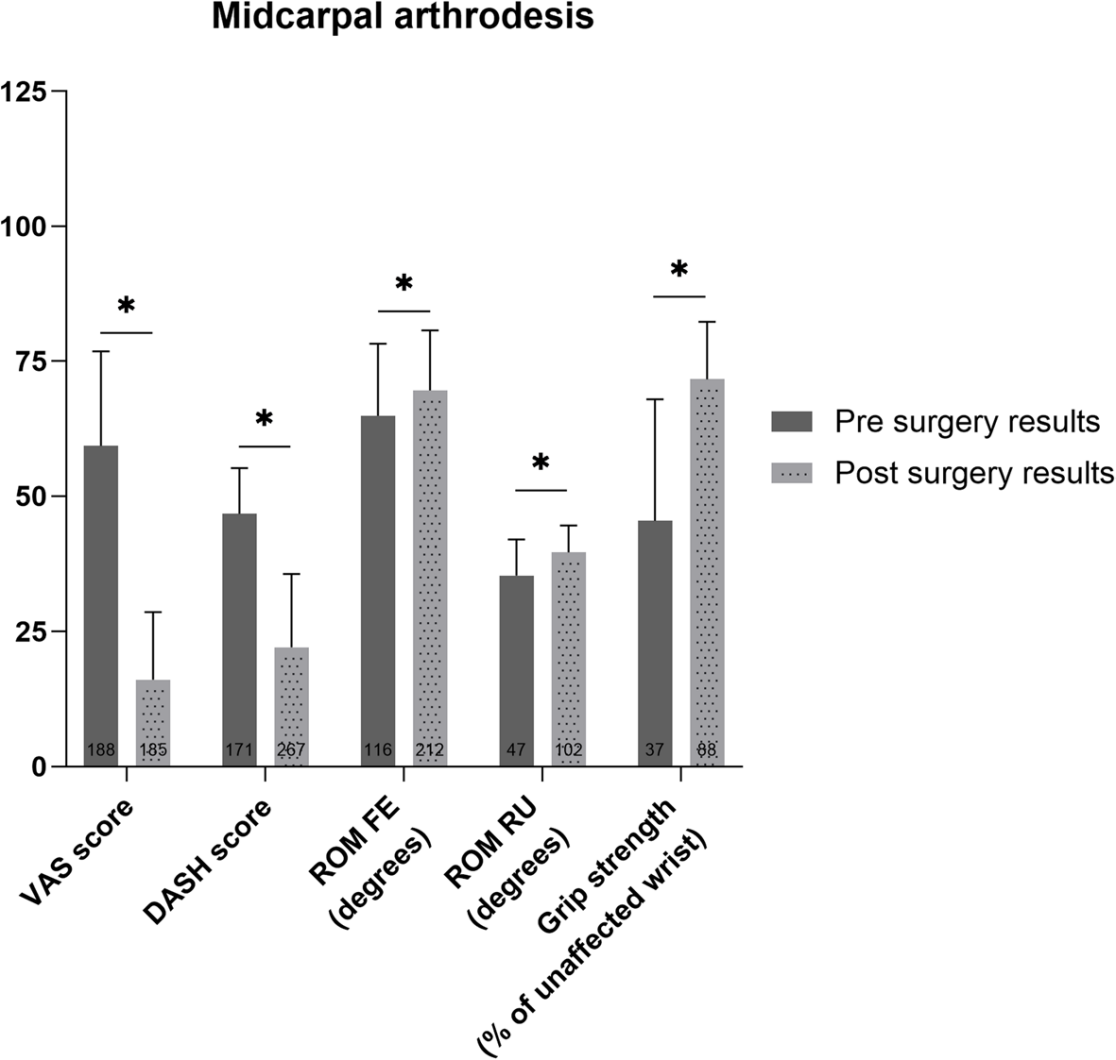
Pre- and postoperative weighted mean (with standard deviation) of patient reported and functional outcome measures for patients who underwent midcarpal arthrodesis. Number of patients on which the mean is calculated is depicted in the base of each bar

The weighted mean of all patient reported and functional outcomes improved after midcarpal arthrodesis (*p*<0.01) (Fig. 6). The difference between pre-and postoperative VAS score, DASH score and grip strength was clinically relevant.

### Radiocarpal arthrodesis

**Fig. 7:**
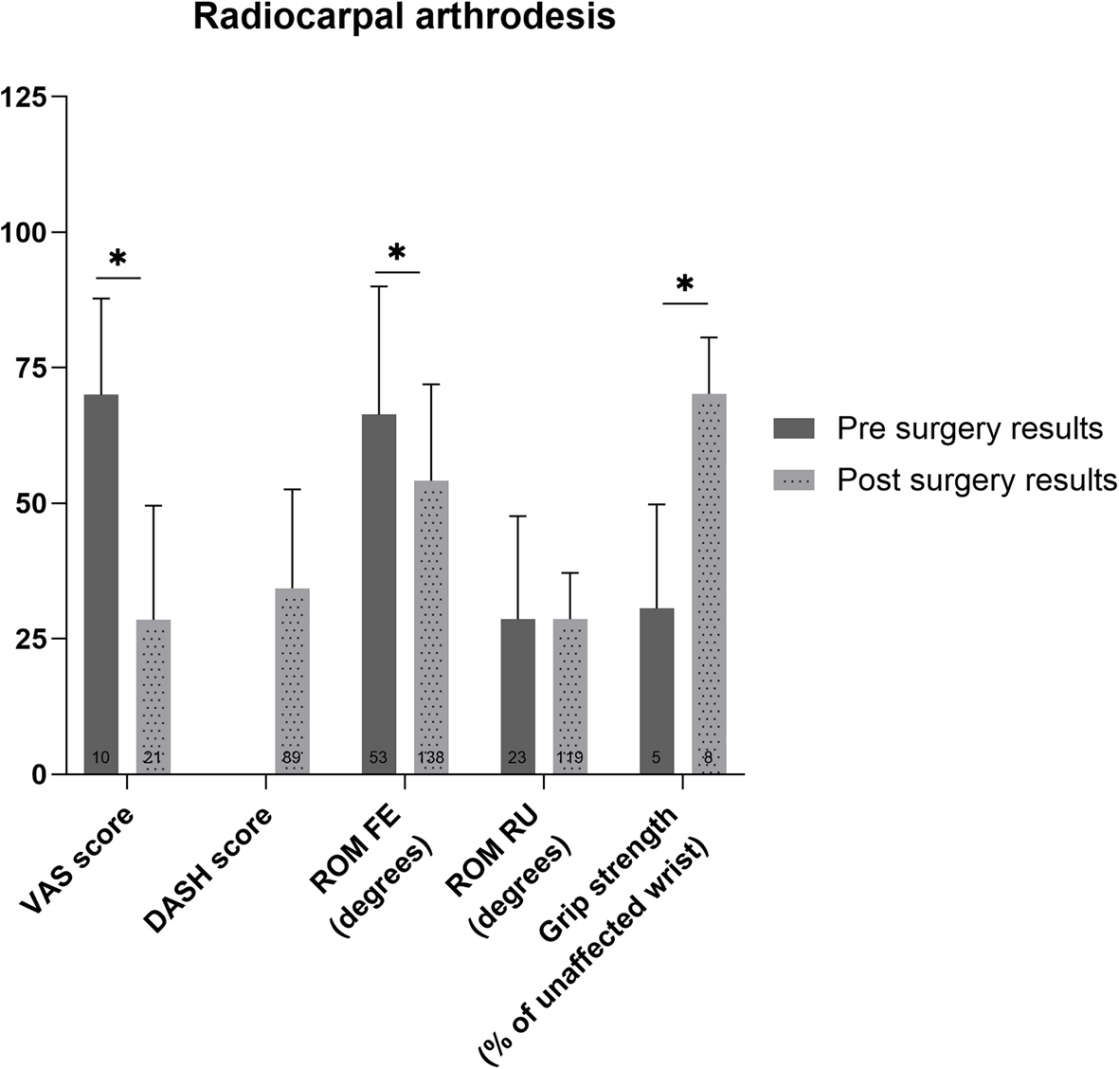
Pre- and postoperative weighted mean (with standard deviation) of patient reported and functional outcome measures for patients who underwent radiocarpal arthrodesis. Number of patients on which the mean is calculated is depicted in the base of each bar

The weighted mean VAS score, ROM FE decreased and the grip strength increased after radiocarpal arthrodesis (*p*<0.01) (Fig. 7). The improvement between pre- and postoperative VAS score and grip strength were both considered clinically relevant. Furthermore, the ROM RU showed no significant change after surgery (*p*=1.00). There were no preoperative DASH scores reported.

### Total arthrodesis

There was only one study that reported the postoperative DASH score of patients after total arthrodesis (45.2 ±22.01). There are no other patient reported and functional outcomes available.

### Sub-analysis

**Fig. 8:**
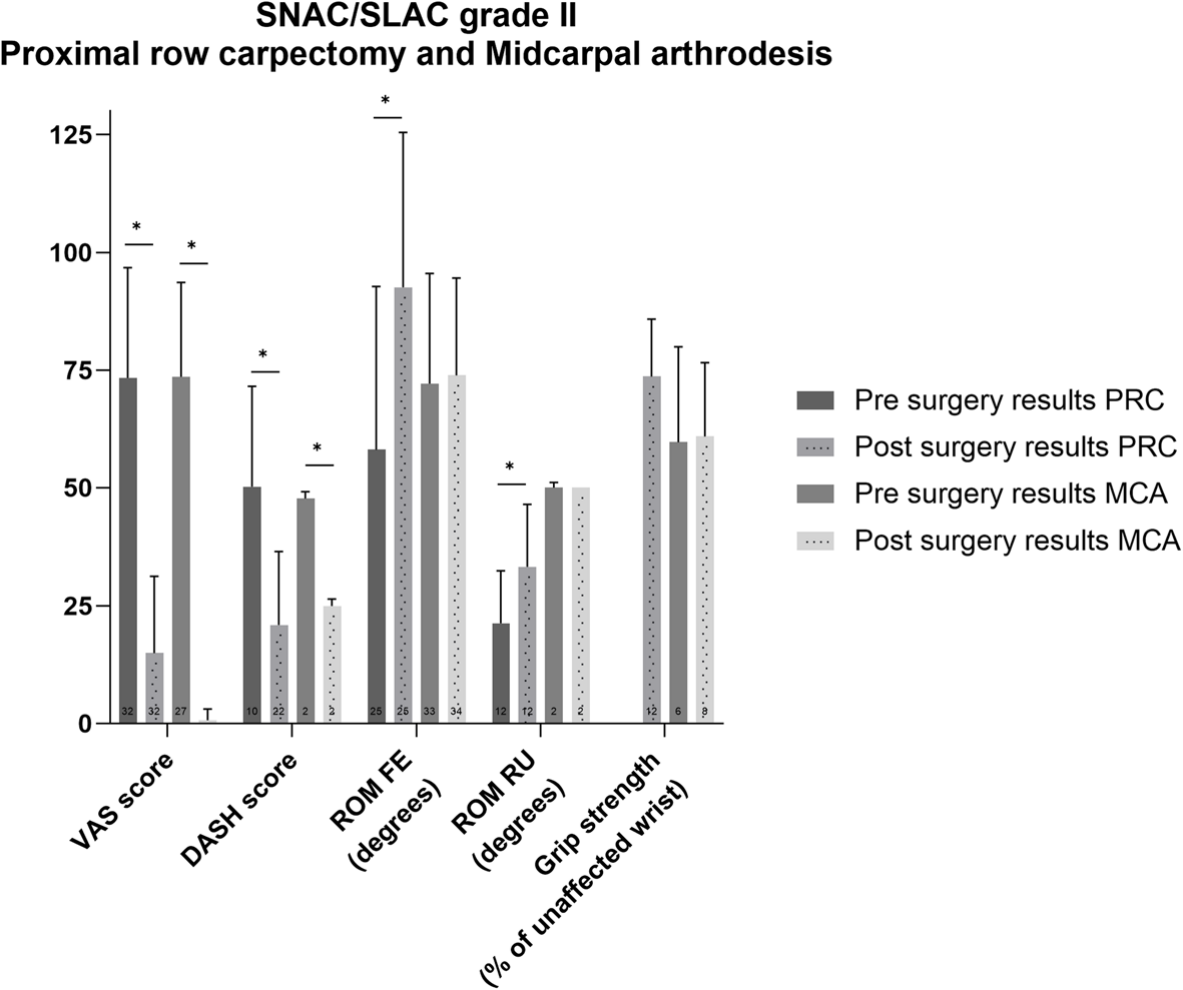
Pre- and postoperative weighted mean (with standard deviation) of patient reported and functional outcome measures for patients suffering from SNAC and SLAC grade II who underwent proximal row carpectomy or midcarpal arthrodesis. Number of patients on which the mean is calculated is depicted in the base of each bar

Finally, the sub-analysis of patient reported and functional outcomes for patients with SNAC or SLAC grade II undergoing proximal row carpectomy or midcarpal arthrodesis are presented in Fig. 8. Weighted mean VAS and DASH score improved (*p*<0.01) after both procedures and improvements were also clinically relevant. Weighted mean ROM improved significantly more after proximal row carpectomy (*p*<0.02) in 25 patients. Preoperative results for the grip strength were not reported. ROM and grip strength did not differ after midcarpal arthrodesis (*p*>0.05).

The pooled pre- and postoperative data of the sub-analysis for the four and five included studies for proximal row carpectomy and midcarpal arthrodesis respectively can be found in Additional Table S5.

## Discussion

This systematic review critically appraises the available evidence of functional and patient reported outcomes for the most common surgical interventions for posttraumatic osteoarthritis of the wrist. The included main interventions were denervation, proximal row carpectomy, midcarpal arthrodesis, radiocarpal arthrodesis, total wrist arthrodesis, total wrist arthroplasty and excisional radiocarpal interposition arthroplasty. A plethora of different techniques are available for each of the main interventions. For example, in midcarpal arthrodeses, two, three or four different carpal bones can be fused. Moreover, the fixation technique for fusion differs from K-wire fixation to screw fixation to plate and screw fixation. Given the large heterogeneity in surgical techniques and the low patient numbers in most series, we pooled the different techniques of each of the stated main interventions. As a result of this systematic review, specific considerations related to various treatments of posttraumatic osteoarthritis of the wrist were identified.

In the included studies covering denervation, patients underwent either a total denervation or a partial denervation of the wrist. A previously performed systematic review by Smeraglia et al. concluded that both partial and total wrist denervation are safe and reliable procedures that provide substantial pain relief and preserve wrist motion [71]. In addition, both Boeckstyns et al. and Schmidt showed pain relief in over 70% of the patients with both non- and posttraumatic osteoarthritis [10, 72]. However, our review revealed no decrease in pain scores after denervation. The ROM in FE and RU did decrease in contrast to previous studies. The contrasting VAS and ROM scores between our study and the previously mentioned studies could be due to our strict selection of high quality studies which included posttraumatic patients only. For instance, many of the studies included in the review of Smeraglia, Boeckstyns and Schmidt were of low quality with a significant number of patients included with other pathologies than posttraumatic osteoarthritis. For example, denervations were performed in patients with Kienbock’s disease, patients with residual pain after partial arthrodesis, idiopathic wrist pain or after sprains of the wrist joint. The reported outcome measurements were not separately reported for the posttraumatic patient cohorts and therefore not included in the current review. Swärd et al. already indicated that posterior and anterior interosseous nerve denervation may not suffice in patients with SNAC/SLAC, since the posterior and anterior interosseous nerve innervate two-thirds of the central part of the joint while the pathological changes due to SNAC/SLAC (even in lower grades) are situated at the radial site of the joint [73]. In contrast to prior studies and based on the results of this review, some reluctance should be advised when considering denervation for patients with posttraumatic osteoarthritis of the wrist.

Both proximal row carpectomy and midcarpal arthrodesis are advocated in patients with SLAC or SNAC grade II. Even though proximal row carpectomy is unsuitable for patients with SNAC or SLAC grade III due to the involvement of the midcarpal joint [74], results from these patients or patients with unspecified SNAC or SLAC grade are often included in meta-analysis and systematic reviews. Including these patients can have an effect on the patient reported and functional outcomes, therefore, we only included patients with SNAC and SLAC grade II. Previous meta-analyses and systematic reviews reported that patient reported and functional outcomes were approximately the same after proximal row carpectomy and midcarpal arthrodesis [13, 15, 74–76]. If a significant difference was found between the two procedures such as in the study of Amer et al., then this difference in favour of proximal row carpectomy was very small (grip strength difference: 1.52%; VAS score difference: 3.0%) ) and not clinically relevant [75]. In order to preserve more grip strength after a PRC, some authors advocate to combine proximal row carpectomy with a resurfacing capitate pyrocarbon implant in order to maintain carpal height [77]. Although a number of series have been published combining proximal row carpectomy with resurfacing capitate pyrocarbon implant, only the study of Szalery et al. met the inclusion criteria of this review.

The subgroup analysis of patients with SLAC or SNAC grade II in this study showed that VAS and DASH scores improved after both procedures.

The FE ROM only improved after proximal row carpectomy. It should be noted that the midcarpal arthrodesis group includes a plethora of different carpal bone fusions by different techniques ranging from four-corner arthrodesis to limited carpal fusion techniques. Although ideally preferable, the existing literature is too scarce and absolute patient numbers are too small to perform a subgroup analysis for each specific technique. Therefore, one should be cautious when interpreting this subgroup analysis as other series found preferable results of limited carpal fusion over proximal row carpectomy [78]. In addition, we were not able to compare the follow-up between the surgical procedures since the exact follow-up of all included patients is not known. However, since some studies suggest that the subjective and functional outcomes after proximal row carpectomy stay stable over time [79, 80], we still think it is a relevant comparison. In our opinion, we prefer a proximal row carpectomy over a midcarpal arthrodesis for patients with a SNAC or SLAC grade II due to a more favourable expected FE ROM. Other results were hard to interpret due to limitations such as small patient numbers and missing data. More comparative research is needed to provide a more definitive conclusion between these surgical procedures.

A radiocarpal arthrodesis is an excellent procedure to reduce pain and improve grip strength in patients with wrist osteoarthritis secondary to a distal radius fracture. Inherent to the procedure, FE ROM is compromised after radiocarpal arthrodesis. In contrast, both FE and RU ROM improve significantly after total wrist arthroplasty. There is insufficient data on grip strength after total wrist arthroplasty. The findings of this review suggest that favorable functional and patient reported outcomes can be expected after a total wrist arthroplasty in comparison to radiocarpal arthrodesis. Although mid-term survival rates of 93-94% in recent series of Reigstad et al. and Holzbauer et al. seem promising [28, 29], long-term outcomes are still unclear. Nevertheless, total wrist arthroplasty could be a safe and reliable option in an older patient population with posttraumatic wrist osteoarthritis.

Several limitations are apparent in this study. First, we found that high quality studies are scarce. Also, the absolute patient numbers that could be included in the systematic review are low, even after pooling all eligible studies. Next, the included studies have a notable heterogeneity in terms of patient characteristics and surgical interventions. For example, the analyzed midcarpal arthrodesis group consisted of different fusions performed with different implants, each having its own theoretical advantages and disadvantages. The heterogeneous patient characteristics make it even more difficult to compare each of the main categorized interventions as it could lead to a potential risk of bias. Lastly, we have to recognize that there is no uniform method to assess patient reported and functional outcomes in current literature and that outcome measures were collected in many ways. This heterogeneity in reporting could also add to a potential risk of bias. More high quality studies and more standardized reporting of patient reported and functional outcomes or the use of national or international registries in wrist surgery would greatly benefit the understanding of the effect of surgeries performed today. This would make it easier to combine the results of smaller (cohort) studies and compare the different surgeries.

## Conclusion

In this systematic review, we performed a thorough, high quality critical appraisal of the available literature regarding the surgical treatment of posttraumatic radiocarpal osteoarthritis. We found that high quality studies are scarce, and when selecting these studies, absolute patient numbers are small. Therefore, some restraint is needed evaluating these results. Evidence of this review did not support the indication for denervation in this particular patient population any more. In patients with SLAC/SNAC II, proximal row carpectomy might be favorable to a midcarpal arthrodesis solely based on better FE ROM of the radiocarpal joint after proximal row carpectomy. Other results were hard to interpret due to limitations such as small patient numbers and missing data. More comparative research is needed to provide a more definitive conclusion between these surgical procedures. In terms of radiocarpal mobility, total wrist arthroplasty might be preferred to radioscapholunate arthrodesis in specific patients with osteoarthritis after a distal radius fracture. The displayed results can help clinicians select the best surgical treatment for each patient and manage the patient’s expectations.

### Supplementary Information

Supplementary Material 1.

Supplementary Material 2.

Supplementary Material 3.

Supplementary Material 4.

Supplementary Material 5.

## Data Availability

All data generated or analysed during this study are included in this published article and its supplementary information files.

## Abbreviations

DASH: Disability of Arm, Shoulder and Hand
FE: flexion/extension
MINORS: Methodological Index for Non-Randomized Studies
PRISMA: Preferred Reporting Items for Systematic review and Meta-Analyses
ROM: Range of motion
RU: radial/ulnar deviation
SLAC: scapholunate advanced collapse
SNAC: scaphoid nonunion advanced collapse
VAS: Visual Analogue Scale
