# Supplementary material for: Patient reported and functional outcome measures after surgical salvage procedures for posttraumatic radiocarpal osteoarthritis – a systematic review
Source: BMC Musculoskelet Disord. 2024 Jun 7;25:453. doi: 10.1186/s12891-024-07527-6 (PMC11157883; doi:10.1186/s12891-024-07527-6)
Supplement: Supplementary file 1 — Supplementary Material 1. [file 12891_2024_7527_MOESM1_ESM.docx]

Additional Table 1: Search strategy

| 1. Pubmed | |
| --- | --- |
| Search number | Query |
| 1 | (posttraumatic) OR (post-traumatic) OR (trauma) OR (trauma*) |
| 2 | (wrist joint[MeSH Terms]) OR (wrist joint) OR (wrist) OR (radiocarpal joint) OR (radiocarpal) |
| 3 | (osteoarthritis[MeSH Terms]) OR (osteoarthritis) OR (OA) OR (degenerative joint disease) OR (joint degeneration) |
| 4 | (scaphoid non union advanced collapse) OR (SNAC) OR (scapholunate advanced collapse) OR (SLAC) OR (scapho* advanced collapse) |
| 5 | #1 AND #2 AND (#3 OR #4) |
| 6 | (surgical procedure, operative[MeSH Terms]) OR (surg* procedure) OR (surgical salvage procedure) OR (surgery) OR (surg*) OR (salvage procedure) |
| 7 | (arthrodesis[MeSH Terms]) OR (arthrodesis) OR (total wrist fusion) OR (TWF) |
| 8 | (partial arthrodesis) |
| 9 | (four corner* arthrodesis) OR (four bone arthrodesis) OR (4 bone arthrodesis) OR (4CA) OR (four corner* fusion) OR (four bone fusion) OR (4 bone fusion) OR (four corner*) OR (4 corner*)OR (four bone) OR (4 bone) OR (corner* arthrodesis) OR (corner* fusion) OR (intercarpal arthrodesis) |
| 10 | (radioscapholunate arthrodesis) OR (radioscapholunate fusion) OR (radiocarpal fusion) OR (RSL) |
| 11 | (proximal row carpectomy) OR (PRC) |
| 12 | (wrist denervation) |
| 13 | (arthroplasty[MeSH Terms]) OR (arthroplasty) OR (total arthroplasty) OR (total replacement) OR (TWA) |
| 14 | (hemiarthroplasty[MeSH Terms]) OR (hemiarthroplasty) OR (partial arthroplasty) OR (PWA) OR (proximal row replacement) |
| 15 | (interposition arthroplasty) OR (pyrocarbon arthroplasty) OR (pyrocarbon implant) |
| 16 | #6 OR #7 OR #8 OR #9 OR #10 OR #11 OR #12 OR #13 OR #14 OR #15 |
| 17 | #5 AND #16 |
| 18 | (functional outcomes) OR (functional testing) OR (functional measurements) OR (functional assessment) |
| 19 | (range of motion, articular[MeSH Terms]) OR (range of motion) OR (ROM) OR (mobility) |
| 20 | (hand strength[MeSH Terms]) OR (hand strength) OR (grip strength) or (hand grip) |
| 21 | ((visual analogue pain scale[MeSH Terms]) OR (VAS) OR (numeric rating scale) OR (NRS) OR (pain[MeSH Terms]) OR (pain) OR (pain assessment[MeSH Terms]) OR (pain assessment)) OR (pain assessment[MeSH Terms]) |
| 22 | (questionnaires[MeSH Terms]) OR (questionnaire) OR (survey) OR (functional outcome questionnaire) OR (patient reported outcome measure) OR (PROM) OR (Disabilities Arm Shoulder Hand) OR (DASH) OR (QDASH) OR (QuickDASH) |
| 23 | #18 OR #19 OR #20 OR #21 OR #22 |
| 24 | #17 AND #23 |

| 1. Embase | |
| --- | --- |
| Search number | Query |
| 1 | (posttraumatic or post-traumatic or trauma or trauma$).mp. |
| 2 | exp wrist/ or (wrist or wrist joint or radiocarpal joint or radiocarpal).mp. |
| 3 | exp osteoarthritis/ or exp joint degeneration/ or (osteoarhtritis or OA or joint degeneration or degenerative joint disease).mp. |
| 4 | (scaphoid non union advanced collapse or SNAC or scapholunate advanced collapse or SLAC or scapho$ advanced collapse).mp. |
| 5 | 1 AND 2 AND (3 OR 4) |
| 6 | exp surgery/ or (surgery or surg$ or surgical procedure or surg$ procedure or surgical salvage procedure or salvage procedure).mp. |
| 7 | exp arthrodesis/ or (arthrodesis or total wrist fusion or TWF).mp. |
| 8 | partial arthrodesis.mp. |
| 9 | (four corner$ arthrodesis or four bone arthrodesis or 4 bone arthrodesis or 4CA or four corner$ fusion or four bone fusion or 4 bone fusion or four corner$ or 4 corner$ or four bone or 4 bone or corner$ arthrodesis or corner$ fusion or intercarpal arthrodesis).mp. |
| 10 | (radioscapholunate arthrodesis or radioscapholunate fusion or radiocarpal fusion or RSL).mp. |
| 11 | (proximal row carpectomy or PRC).mp. |
| 12 | (wrist.mp. and exp denervation/) or wrist denervation.mp. |
| 13 | exp total arthroplasty/ or exp arthroplasty/ or (arthroplasty or total arthroplasty or total replacement or TWA).mp. |
| 14 | exp hemiarthroplasty/ or (hemiarthroplasty or partial arthroplasty or PWA or proximal row replacement).mp. |
| 15 | (interposition arthroplasty or pyrocarbon arthroplasty or pyrocarbon implant).mp. |
| 16 | 6 or 7 or 8 or 9 or 10 or 11 or 12 or 13 or 14 or 15 |
| 17 | 5 and 16 |
| 18 | exp functional assessment/ or (functional assessment or functional outcomes or functional testing or functional measurements).mp. |
| 19 | exp range of motion/ or (range of motion or ROM or mobility).mp. |
| 20 | exp hand strength/ or exp grip strength/ or exp hand grip/ or (hand strength or grip strength or hand grip).mp. |
| 21 | exp numeric rating scale/ or exp pain/ or exp pain assessment/ or (numeric rating scale or NRS or pain or pain assessment or visual analogue pain scale or VAS).mp. |
| 22 | exp questionnaire/ or exp patient-reported outcome/ or (questionnaire or survey or patient-reported outcome or PROM or Disabilities Arm Shoulder Hand or DASH or QDASH or QuickDASH).mp. |
| 23 | 18 or 19 or 20 or 21 or 22 |
| 24 | 17 and 23 |

| 1. Cochrane | |
| --- | --- |
| Search number | Query |
| 1 | (posttraumatic) OR (post-traumatic) OR (trauma) OR (trauma*) (Word variations have been searched) |
| 2 | MeSH descriptor: [Wrist Joint] explode all trees |
| 3 | (wrist joint) OR (wrist) OR (radiocarpal joint) OR (radiocarpal) (Word variations have been searched) |
| 4 | #2 OR #3 |
| 5 | MeSH descriptor: [Osteoarthritis] explode all trees |
| 6 | (osteoarthritis) OR (OA) OR (degenerative joint disease) OR (joint degeneration) (Word variations have been searched) |
| 7 | #5 OR #6 |
| 8 | (scaphoid non union advanced collapse) OR (SNAC) OR (SLAC) OR (joint descapholunate advanced collapsegeneration) AND (scapho* advanced collapse) (Word variations have been searched) |
| 9 | #1 and #4 and (#7 or #8) |
| 10 | MeSH descriptor: [Surgical Procedures, Operative] explode all trees |
| 11 | (surg* procedure) OR (surgical salvage procedure) OR (surgery) OR (surg*) OR (salvage procedure) |
| 12 | #10 or #11 |
| 13 | MeSH descriptor: [Arthrodesis] explode all trees |
| 14 | (arthrodesis) OR (total wrist fusion) OR (TWF) |
| 15 | #13 or #14 |
| 16 | (partial arthrodesis) |
| 17 | (four bone arthrodesis) OR (four corner* arthrodesis) OR (four bone fusion) AND (four corner* fusion):ti,ab,kw |
| 18 | (radioscapholunate arthrodesis) OR (radioscapholunate fusion) OR (radiocarpal fusion) OR (RSL) |
| 19 | (proximal row carpectomy) OR (PRC) |
| 20 | (wrist denervation) |
| 21 | MeSH descriptor: [Arthroplasty] explode all trees |
| 22 | (arthroplasty) OR (total arthroplasty) OR (total replacement) OR (TWA) |
| 23 | #21 or #22 |
| 24 | MeSH descriptor: [Hemiarthroplasty] explode all trees |
| 25 | (hemiarthroplasty) OR (partial arthroplasty) OR (PWA) OR (proximal row replacement) |
| 26 | #24 or #25 |
| 27 | (interposition arthroplasty) OR (pyrocarbon arthroplasty) OR (pyrocarbon implant) |
| 28 | #12 or #15 or #16 or #17 or #18 or #19 or #20 or #23 or #26 or #27 |
| 29 | #9 and #28 |
| 30 | (functional outcomes) OR (functional testing) OR (functional measurements) OR (functional assessment) |
| 31 | MeSH descriptor: [Hand Strength] explode all trees |
| 32 | (hand strength) OR (grip strength) OR (hand grip) |
| 33 | #31 or #32 |
| 34 | MeSH descriptor: [Range of Motion, Articular] explode all trees |
| 35 | (range of motion) OR (ROM) OR (mobility) |
| 36 | #34 or #35 |
| 37 | MeSH descriptor: [Pain Measurement] explode all trees |
| 38 | MeSH descriptor: [Pain] explode all trees |
| 39 | (pain assessment) OR (visual analogue pain scale) OR (VAS) OR (Numeric rating scale) OR (NRS) |
| 40 | #37 or #38 or #39 |
| 41 | MeSH descriptor: [Surveys and Questionnaires] explode all trees |
| 42 | MeSH descriptor: [Patient Reported Outcome Measures] explode all trees |
| 43 | (questionnaire) OR (survey):kw OR (functional outcome questionnaire) OR (patient reported outcome measure) OR (PROM) |
| 44 | (Disabilities arm shoulder hand) OR (DASH) OR (QuickDASH) |
| 45 | #41 or #42 or #43 or #44 |
| 46 | #30 or #33 or #36 or #40 or #45 |
| 47 | #29 and #46 |
