# Supplementary material for: Patient reported and functional outcome measures after surgical salvage procedures for posttraumatic radiocarpal osteoarthritis – a systematic review
Source: BMC Musculoskelet Disord. 2024 Jun 7;25:453. doi: 10.1186/s12891-024-07527-6 (PMC11157883; doi:10.1186/s12891-024-07527-6)
Supplement: Supplementary file 2 — Supplementary Material 2. [file 12891_2024_7527_MOESM2_ESM.docx]

Additional Table 2: MINORS score of included studies divided per surgical salvage procedure

| Study | 1 | 2 | 3 | 4 | 5 | 6 | 7 | 8 | Total |
| --- | --- | --- | --- | --- | --- | --- | --- | --- | --- |
| Denervation | | | | | | | | | |
| Radu et al. 2010 | 2 | 0 | 0 | 2 | 2 | 2 | 0 | 0 | 8 |
| Rothe et al. 2006 | 2 | 2 | 2 | 2 | 2 | 2 | 1 | 0 | 13 |
| Schweizer et al. 2006 | 2 | 2 | 0 | 2 | 2 | 2 | 1 | 0 | 11 |
| Weinstein et al. 2002 | 2 | 2 | 0 | 2 | 2 | 2 | 2 | 0 | 12 |
| Interposition arthroplasty | | | | | | | | | |
| Pequignot et al. 2000 | 2 | 2 | 0 | 2 | 2 | 2 | 1 | 0 | 11 |
| Szalay et al. 2011 | 2 | 2 | 0 | 2 | 2 | 2 | 2 | 0 | 12 |
| Total arthroplasty | | | | | | | | | |
| Holzbauer et al. 2022 | 2 | 2 | 2 | 2 | 2 | 2 | 1 | 0 | 13 |
| Reigstad et al. 2012 | 2 | 2 | 2 | 2 | 2 | 2 | 1 | 0 | 13 |
| Proximal row carpectomy | | | | | | | | | |
| Aita et al. 2016 | 2 | 2 | 2 | 2 | 2 | 2 | 2 | 0 | 14 |
| Cohen et al. 2001 | 2 | 2 | 2 | 2 | 2 | 2 | 0 | 0 | 12 |
| Jebson et al. 2003 | 2 | 2 | 0 | 2 | 2 | 2 | 1 | 0 | 11 |
| Nagelvoort et al. 2002 | 2 | 2 | 0 | 2 | 2 | 2 | 1 | 0 | 11 |
| Pogliacomi et al. 2014 | 0 | 2 | 0 | 2 | 2 | 2 | 2 | 0 | 10 |
| Salamon et al. 1996 | 2 | 2 | 2 | 2 | 2 | 2 | 1 | 0 | 13 |
| De Smet et al. 2005 | 2 | 2 | 2 | 2 | 2 | 2 | 1 | 0 | 13 |
| Streich et al. 2008 | 2 | 2 | 2 | 2 | 2 | 2 | 2 | 0 | 14 |
| Midcarpal arthrodesis | | | | | | | | | |
| Abdelaziz et al. 2020 | 2 | 2 | 2 | 2 | 2 | 2 | 2 | 0 | 14 |
| Aita et al. 2016 | 2 | 2 | 2 | 2 | 2 | 2 | 2 | 0 | 14 |
| Calundruccio et al. 2000 | 2 | 2 | 0 | 2 | 2 | 2 | 2 | 0 | 12 |
| Cha et al. 2013 | 2 | 2 | 0 | 2 | 2 | 2 | 2 | 0 | 12 |
| Chung et al. 2016 | 2 | 2 | 2 | 2 | 2 | 2 | 1 | 0 | 13 |
| Cohen et al. 2001 | 2 | 2 | 2 | 2 | 2 | 2 | 0 | 0 | 12 |
| Le Corre et al. 2015 | 2 | 2 | 0 | 2 | 2 | 2 | 1 | 0 | 11 |
| Dimitrios et al. 2010 | 2 | 2 | 2 | 2 | 2 | 2 | 2 | 0 | 14 |
| Durand et al. 2007 | 2 | 2 | 2 | 2 | 2 | 2 | 0 | 0 | 12 |
| Ferreres et al. 2009 | 2 | 2 | 0 | 2 | 2 | 2 | 1 | 0 | 11 |
| Ghargozloo et al. 2002 | 2 | 2 | 2 | 2 | 2 | 2 | 2 | 0 | 14 |
| Hernekamp et al. 20016 | 2 | 2 | 0 | 2 | 2 | 2 | 1 | 0 | 11 |
| Huang et al. 2021 | 2 | 2 | 2 | 2 | 2 | 2 | 2 | 0 | 14 |
| Kendal et al. 2005 | 2 | 2 | 2 | 2 | 2 | 2 | 1 | 0 | 13 |
| Khan et al. 2013 | 2 | 2 | 0 | 2 | 2 | 2 | 2 | 0 | 12 |
| Maire et al. 2011 | 2 | 2 | 2 | 2 | 2 | 2 | 2 | 0 | 14 |
| Mantovani et al. 2010 | 2 | 2 | 2 | 2 | 2 | 2 | 2 | 0 | 14 |
| Schindelar et al. 2022 | 2 | 2 | 2 | 2 | 2 | 2 | 2 | 0 | 14 |
| Singh et al. 2015 | 2 | 2 | 0 | 2 | 2 | 2 | 2 | 0 | 12 |
| De Smet et al. 2006 | 2 | 0 | 0 | 2 | 2 | 2 | 2 | 0 | 10 |
| De Smet et al. 2009 | 2 | 2 | 0 | 2 | 2 | 2 | 2 | 0 | 12 |
| Tielemans et al. 2017 | 2 | 2 | 0 | 2 | 2 | 2 | 1 | 0 | 11 |
| Undurraga et al. 2021 | 2 | 2 | 0 | 2 | 2 | 2 | 2 | 0 | 12 |
| Winkler et al. 2010 | 2 | 0 | 0 | 2 | 2 | 2 | 0 | 0 | 8 |
| Yao et al. 2017 | 2 | 2 | 2 | 2 | 2 | 2 | 1 | 0 | 13 |
| Radiocarpal arthrodesis | | | | | | | | | |
| Bach et al. 1991 | 1 | 2 | 0 | 2 | 2 | 2 | 2 | 0 | 11 |
| Beyermann et al. 2000 | 2 | 2 | 2 | 2 | 2 | 2 | 1 | 0 | 13 |
| Degeorge et al. 2020 | 2 | 2 | 0 | 2 | 2 | 2 | 1 | 0 | 11 |
| Garcia-Elias et al. 2005 | 2 | 0 | 0 | 2 | 2 | 2 | 2 | 0 | 10 |
| Inoue et al. 1992 | 2 | 2 | 0 | 2 | 2 | 2 | 1 | 0 | 11 |
| Kilgus et al. 2003 | 2 | 0 | 0 | 2 | 2 | 2 | 2 | 0 | 10 |
| Quadbauer et al. 2017 | 2 | 2 | 0 | 2 | 2 | 2 | 1 | 0 | 11 |
| Tomaino et al. 1994 | 2 | 2 | 0 | 2 | 2 | 2 | 2 | 0 | 12 |
| Yajima et al. 1994a | 2 | 2 | 2 | 2 | 2 | 2 | 2 | 0 | 14 |
| Yajima et al. 1994b | 2 | 2 | 1 | 2 | 2 | 2 | 2 | 0 | 13 |
| Total arthrodesis | | | | | | | | | |
| De Smet et al. 2006 | 2 | 2 | 0 | 2 | 2 | 2 | 2 | 0 | 12 |
