# Supplementary material for: Patient reported and functional outcome measures after surgical salvage procedures for posttraumatic radiocarpal osteoarthritis – a systematic review
Source: BMC Musculoskelet Disord. 2024 Jun 7;25:453. doi: 10.1186/s12891-024-07527-6 (PMC11157883; doi:10.1186/s12891-024-07527-6)
Supplement: Supplementary file 3 — Supplementary Material 3. [file 12891_2024_7527_MOESM3_ESM.docx]

Additional Table 3: Diagnosis of patients per surgical salvage procedure

|  | SNAC/ SLAC | SNAC unspecified | SNAC II | SNAC III | SNAC IV | SLAC unspecified | SLAC II | SLAC III | SLAC IV | DRF | SLD | SN | Other |
| --- | --- | --- | --- | --- | --- | --- | --- | --- | --- | --- | --- | --- | --- |
| Denervation | 11 | 25 | 18 | 18 | 0 | 8 | 10 | 5 | 9 | 31 | 0 | 0 | 0 |
| Interposition arthroplasty | 0 | 1 | 8 | 3 | 0 | 4 | 3 | 2 | 0 | 0 | 0 | 0 | 0 |
| Total arthroplasty | 0 | 13 | 0 | 8 | 8 | 11 | 0 | 7 | 7 | 8 | 0 | 0 | 0 |
| Proximal row carpectomy | 0 | 29 | 26 | 0 | 0 | 5 | 28 | 8 | 0 | 0 | 0 | 16 | 2 |
| Midcarpal arthrodesis | 38 | 21 | 55 | 74 |  | 52 | 51 | 133 | 0 | 0 | 0 | 0 | 1 |
| Radiocarpal arthrodesis | 0 | 0 | 2 | 0 | 0 | 0 | 0 | 0 | 0 | 118 | 9 | 1 | 8 |
| Total arthrodesis | 0 | 5 | 0 | 0 | 0 | 14 | 0 | 0 | 0 | 0 | 0 | 0 | 0 |

_SNAC: scaphoid nonunion advanced collapse; SLAC: scapholunate advanced collapse; DRF: distal radial fracture; SLD: scapholunate dissociation; SN: scaphoid non-union_
