# Supplementary material for: Patient reported and functional outcome measures after surgical salvage procedures for posttraumatic radiocarpal osteoarthritis – a systematic review
Source: BMC Musculoskelet Disord. 2024 Jun 7;25:453. doi: 10.1186/s12891-024-07527-6 (PMC11157883; doi:10.1186/s12891-024-07527-6)
Supplement: Supplementary file 4 — Supplementary Material 4. [file 12891_2024_7527_MOESM4_ESM.docx]

Additional Table 4: Weighted pre and post surgery mean of patient reported and functional outcomes per surgical salvage procedure

|  | Pre surgery | | | | | Post surgery | | | | | |  |
| --- | --- | --- | --- | --- | --- | --- | --- | --- | --- | --- | --- | --- |
|  | N articles | N wrist | Weighted mean | Estimated standard deviation | Confidence interval | | N articles | N wrist | Weighted mean | Estimated standard deviation | Confidence interval | P-value |
| Denervation | | | | | | | | | | | |  |
| VAS score | 1 | 30 | 70.4 | 30.2 | 11.3 | | 1 | 30 | 60.8 | 20.9 | 7.8 | 0.16 |
| DASH score | NR | NR | NR | NR | NR | | 3 | 84 | 30.0 | 18.1 | 3.9 | - |
| ROM FE | 2 | 76 | 86.4 | 23.5 | 5.4 | | 2 | 76 | 77.3 | 23.8 | 5.4 | 0.02 |
| ROM RU | 1 | 46 | 35.4 | 12.2 | 3.6 | | 1 | 46 | 29.2 | 9.7 | 2.9 | 0.01 |
| Interposition arthroplasty | | | | | | | | | | | |  |
| VAS score | 1 | 5 | 61.2 | 5.4 | 6.8 | | 1 | 21 | 14.3 | 3.0 | 1.4 | <0.01 |
| DASH score | 1 | 5 | 51.3 | 4.2 | 5.3 | | 1 | 5 | 8.0 | 6.2 | 7.7 | <0.01 |
| ROM FE | 1 | 5 | 54.0 | 7.6 | 9.5 | | 2 | 21 | 84.4 | 11.4 | 5.2 | <0.01 |
| ROM RU | 1 | 5 | 28.0 | 5.9 | 7.4 | | 1 | 5 | 32.0 | 3.2 | 3.9 | 0.22 |
| Grip strength | 1 | 5 | 70.0 | 4.3 | 5.4 | | 2 | 21 | 84.1 | 15.7 | 7.1 | <0.01 |
| Total arthroplasty | | | | | | | | | | | |  |
| VAS score | 2 | 53 | 69.8 | 1.4 | 0.4 | | 2 | 53 | 28.2 | 2.5 | 0.7 | <0.01 |
| DASH score | 2 | 62 | 56.4 | 14.7 | 3.7 | | 2 | 53 | 21.8 | 18.9 | 5.2 | <0.01 |
| ROM FE | 1 | 32 | 48.7 | 15.0 | 5.4 | | 1 | 32 | 66.8 | 12.9 | 4.6 | <0.01 |
| ROM RU | 1 | 32 | 22.6 | 7.0 | 2.5 | | 1 | 32 | 36.0 | 8.5 | 3.1 | <0.01 |
| Proximal row carpectomy | | | | | | | | | | | |  |
| VAS score | 2 | 32 | 73.3 | 23.5 | 8.5 | | 3 | 35 | 15.0 | 18.5 | 6.4 | <0.01 |
| DASH score | 1 | 12 | 51.9 | 24.2 | 15.4 | | 4 | 53 | 18.0 | 13.5 | 4.5 | <0.01 |
| ROM FE | 5 | 56 | 72.1 | 35.0 | 9.4 | | 5 | 56 | 80.3 | 19.9 | 5.3 | 0.13 |
| ROM RU | 2 | 30 | 32.4 | 11.4 | 4.3 | | 4 | 43 | 36.4 | 9.8 | 3.0 | 0.12 |
| Grip strength | NR | NR | NR | NR | NR | | 4 | 59 | 76.2 | 22.8 | 5.9 | - |
| Midcarpal arthrodesis | | | | | | | | | | | |  |
| VAS score | 9 | 188 | 59.4 | 17.5 | 2.5 | | 9 | 185 | 16.1 | 12.5 | 1.8 | <0.01 |
| DASH score | 8 | 171 | 46.7 | 8.5 | 1.4 | | 13 | 267 | 22.1 | 13.6 | 1.6 | <0.01 |
| ROM FE | 10 | 116 | 64.9 | 13.4 | 2.5 | | 17 | 212 | 69.6 | 11.1 | 1.5 | <0.01 |
| ROM RU | 4 | 47 | 35.4 | 6.6 | 1.9 | | 8 | 102 | 39.7 | 4.9 | 1.0 | <0.01 |
| Grip strength | 3 | 37 | 45.4 | 22.5 | 7.5 | | 7 | 88 | 71.7 | 10.7 | 2.3 | <0.01 |
| Radiocarpal arthrodesis | | | | | | | | | | | |  |
| VAS score | 1 | 10 | 70.0 | 17.8 | 12.7 | | 2 | 21 | 28.5 | 21.1 | 9.6 | <0.01 |
| DASH score | NR | NR | NR | NR | NR | | 4 | 89 | 34.3 | 18.2 | 3.8 | - |
| ROM FE | 6 | 53 | 66.4 | 23.7 | 6.5 | | 10 | 138 | 54.1 | 17.9 | 3.0 | <0.01 |
| ROM RU | 2 | 23 | 28.6 | 19.0 | 8.2 | | 7 | 119 | 28.6 | 8.5 | 1.6 | 1.00 |
| Grip strength | 1 | 5 | 30.6 | 19.2 | 23.8 | | 2 | 8 | 70.2 | 10.4 | 8.7 | <0.01 |
| Total arthroplasty | | | | | | | | | | | |  |
| DASH score | NR | NR | NR | NR | NR | | 1 | 19 | 45.2 | 22.0 | 10.6 | - |
