# Supplementary material for: Patient reported and functional outcome measures after surgical salvage procedures for posttraumatic radiocarpal osteoarthritis – a systematic review
Source: BMC Musculoskelet Disord. 2024 Jun 7;25:453. doi: 10.1186/s12891-024-07527-6 (PMC11157883; doi:10.1186/s12891-024-07527-6)
Supplement: Supplementary file 5 — Supplementary Material 5. [file 12891_2024_7527_MOESM5_ESM.docx]

Additional Table 5: Weighted pre and post surgery mean of patient reported and functional outcomes for patients suffering from SNAC and SLAC grade II who underwent proximal row carpectomy or midcarpal arthrodesis

|  | Pre surgery | | | | | Post surgery | | | | |  |
| --- | --- | --- | --- | --- | --- | --- | --- | --- | --- | --- | --- |
|  | N articles | N wrist | Weighted mean | Estimated standard deviation | Confidence interval | N articles | N wrist | Weighted mean | Estimated standard deviation | Confidence interval | P-value |
| Proximal row carpectomy | | | | | | | | | | |  |
| VAS score | 2 | 32 | 73.3 | 23.4 | 8.5 | 2 | 32 | 15.0 | 16.3 | 5.4 | <0.01 |
| DASH score | 1 | 10 | 50.2 | 21.3 | 15.3 | 2 | 22 | 20.9 | 15.6 | 6.9 | <0.01 |
| ROM FE | 2 | 25 | 58.1 | 34.7 | 14.3 | 2 | 25 | 92.6 | 32.9 | 13.6 | <0.01 |
| ROM RU | 1 | 12 | 21.3 | 11.1 | 7.1 | 1 | 12 | 33.3 | 13.2 | 8.4 | 0.02 |
| Grip strength | NR | NR | NR | NR | NR | 1 | 12 | 73.6 | 12.2 | 7.8 | - |
| Midcarpal arthrodesis | | | | | | | | | | |  |
| VAS score | 3 | 27 | 73.6 | 20.1 | 7.9 | 3 | 26 | 0.7 | 2.4 | 1.0 | <0.01 |
| DASH score | 1 | 2 | 47.7 | 1.5 | 13.3 | 1 | 2 | 25.0 | 1.5 | 13.3 | <0.01 |
| ROM FE | 4 | 33 | 72.1 | 23.5 | 8.3 | 5 | 34 | 73.9 | 20.6 | 7.2 | 0.74 |
| ROM U | 1 | 2 | 50.0 | 1.1 | 9.8 | 1 | 2 | 50.0 | 0 | 0 | 1.00 |
| Grip strength | 1 | 6 | 59.1 | 20.2 | 21.2 | 2 | 8 | 60.9 | 15.7 | 13.1 | 0.91 |
